# Novel Functional Genes Involved in Transdifferentiation of Canine ADMSCs Into Insulin-Producing Cells, as Determined by Absolute Quantitative Transcriptome Sequencing Analysis

**DOI:** 10.3389/fcell.2021.685494

**Published:** 2021-06-28

**Authors:** Pengxiu Dai, Jiakai Li, Yijing Chen, Luwen Zhang, Xinke Zhang, Jinglu Wang, Guixiang Qi, Yihua Zhang

**Affiliations:** Shaanxi Branch of National Stem Cell Engineering and Technology Centre, College of Veterinary Medicine, Northwest A&F University, Yangling, China

**Keywords:** adipose-derived mesenchymal stem cells, transdifferentiation, insulin producing cells, novel genes, Absolute Quantitative Transcriptome Sequencing Analysis

## Abstract

The transdifferentiation of adipose-derived mesenchymal stem cells (ADMSCs) into insulin-producing cells (IPCs) is a potential resource for the treatment of diabetes. However, the changes of genes and metabolic pathways on the transdifferentiation of ADMSCs into IPCs are largely unknown. In this study, the transdifferentiation of canine ADMSCs into IPCs was completed using five types of procedures. Absolute Quantitative Transcriptome Sequencing Analysis was performed at different stages of the optimal procedure. A total of 60,151 transcripts were obtained. Differentially expressed genes (DEGs) were divided into five groups: IPC1 vs. ADSC (1169 upregulated genes and 1377 downregulated genes), IPC2 vs. IPC1 (1323 upregulated genes and 803 downregulated genes), IPC3 vs. IPC2 (722 upregulated genes and 680 downregulated genes), IPC4 vs. IPC3 (539 upregulated genes and 1561 downregulated genes), and Beta_cell vs. IPC4 (2816 upregulated genes and 4571 downregulated genes). The gene ontology (GO) and Kyoto Encyclopedia of Genes and Genomes (KEGG) enrichment analysis of DEGs revealed that many genes and signaling pathways that are essential for transdifferentiation. *Hnf1B*, *Dll1*, *Pbx1*, *Rfx3*, and *Foxa1* were screened out, and the functions of five genes were verified further by overexpression and silence. *Foxa1*, *Pbx1*, and *Rfx3* exhibited significant effects, can be used as specific key regulatory factors in the transdifferentiation of ADMSCs into IPCs. This study provides a foundation for future work to understand the mechanisms of the transdifferentiation of ADMSCs into IPCs and acquire IPCs with high maturity.

## Introduction

Diabetes encompasses a group of lifelong metabolic diseases, and common drug therapies are not able to cure it. Long-term use of drugs and the continuous injection of insulin greatly reduce the patient’s quality of life, and strengthening of treatment increases risk of hypoglycemic coma and can even be life-threatening (Cattin, 2016; Harreiter and Roden, 2019; Petersmann et al., 2019). Thus, a safe and effective treatment for diabetes and its complications is urgently needed. At first, islet transplantation was considered as an excellent approach for curing diabetes, but its clinical application was greatly limited by lack of islet donor sources, low islet survival rate *in vitro* and immune rejection after transplantation (Bruni et al., 2014; Gamble et al., 2018; Rickels and Robertson, 2019). Therefore, the search for insulin-producing cells (IPCs) from other sources to replace islet transplantation has become an active area of research.

Adipose-derived mesenchymal stem cells (ADMSCs) are abundant in sources, are easily isolated and cultivated, exhibit pluripotent differentiation potential and show low immunogenicity after transplantation, serving as ideal seed cells for the treatment of diabetes and its complications (Kim et al., 2010; Zhang et al., 2016; Takemura et al., 2019; Wada and Ikemoto, 2019; Tokuda et al., 2020). Numerous small molecule compounds, growth factors, activators, and inhibitors can transdifferentiate ADMSCs into IPCs and improve IPCs survival and ability to release insulin *in vitro* (Dayer et al., 2017; Anjum et al., 2018; Ikemoto et al., 2018; Shahjalal et al., 2018; Pavathuparambil Abdul Manaph et al., 2019; Ghoneim et al., 2020). The overexpression of *Pdx1*, *Neurog3*, *MafA*, and *Pax4* can improve transdifferentiation efficiency and insulin secretion (Limbert et al., 2011; Xu et al., 2017; Zhu et al., 2017; Dayer et al., 2019). However, various transdifferentiation methods have not been systematically compared with one another, leaving various methods in a chaotic state, and opinions on transdifferentiation efficiency vary. Moreover, the changes of genes and metabolic pathways on the transdifferentiation of ADMSCs into IPCs is largely unknown. Thus, ADMSCs transdifferentiate into functional mature beta cells need more research.

In this study, canine ADMSCs were transdifferentiated into IPCs by five types of procedures, the optimal procedure was determined through comparison. Absolute Quantitative Transcriptome Sequencing Analysis was performed at different stages of the optimal procedure to study the changes in genes and metabolic pathways during the transdifferentiation process for the first time. The datasets obtained provided important reference value for the study of the transdifferentiation of ADMSCs into IPCs, islet development and canine genes pool. Five functional genes were screened out. The functions of these genes were verified by overexpression and silencing. This study provides a foundation for future work to understand the mechanisms of the transdifferentiation of ADMSCs into IPCs and acquire IPCs with high maturity.

## Materials and Methods

### Animal

All the dogs (Beagle, Female, 2–5 years old) were purchased from Northwest Agriculture and Forestry University Animal Laboratories (Xian, China). All of the dogs were reared, obtained, and housed in accordance with our institute’s laboratory animal requirements, the dogs were kept in cages in a feeding room without purification equipment at a temperature of 18–25°C, humidity of 40–60%, airflow value of 0.13–0.18 m/s, ventilation rate of 10–20 times per hour, light normal, noise below 60 dB, and all procedures and the study design were conducted in accordance with the Guide for the Care and Use of Laboratory Animals (Ministry of Science and Technology of China, 2006) and were approved by the Animal Ethical and Welfare Committee of Northwest Agriculture and Forest University (Approval No: 2020002).

### Isolation and Culture of Canine ADMSCs

Canine inguinal adipose tissue was obtained by aseptic surgery. The adipose tissue was minced using a sterile scissors and placed in a 50-mL sterile tube with triple volume of 0.1% type I collagenase (Sigma, Ronkonkoma, NY, United States) solution, the tube was transferred to shaker at 180 r/min, 37 °C for 60 min (Raposio et al., 2017). α-MEM Medium [MEM Alpha Modification Medium (Gibco, Waltham, MA, United States) supplemented with 10% fetal bovine serum (Zeta Life, Menlo Park, CA, United States), 100 U/mL penicillin (Sigma, Ronkonkoma, NY, United States), 0.1 mg/mL streptomycin (Sigma, Ronkonkoma, NY, United States) and 0.5 μg/mL Mycoplasma Removal Agent (MP Biomedicals, Irvine, CA, United States)] was used to stop digestion, the tube was centrifuged at 1000 r/min for 5 min, and the upper suspension and floating fat were discarded (Zuk et al., 2001; Vieira et al., 2010). Cells were resuspended in the α-MEM Medium, the suspension was filtered by 200-mesh sieves and centrifuged at 1000 r/min for 5 min, and the supernatant was discarded. Cells were resuspended in α-MEM Medium, transferred to a 60-mm cell culture dish (ThermoFisher Scientific, Waltham, MA, United States), and cultured in a carbon dioxide incubator at 37 °C, 5% CO2 (Palumbo et al., 2015). When the cells grew to 90%, they were digested with trypsin (Gibco, Waltham, MA, United States) and passaged at a ratio of 1:3.

### Identification of Canine ADMSCs

The fourth-generation canine ADMSCs were inoculated into 96-well plates at 5 × 10^2^ cells per well for a total of 44 wells. Contents were taken from four wells every day, and the MTT Cell Proliferation Assay Kit (ThermoFisher Scientific, Waltham, MA, United States) was used to determine the proliferation of cells with continuous determination for 11 days.

The fourth-generation canine ADMSCs were adjusted to 2 × 10^6^ cells/mL suspension; 100 μL cell suspension was transferred into a flow cytometry tube and 5 μL CD13-FITC, CD29-FITC, CD31-FITC, CD44-FITC, CD45-PE, CD73-FITC, CD90-PE, CD105-PE, and CD235a-FITC fluorescent antibodies (BD Biosciences, San Jose, CA, United States) were added. Cells were incubated without light for 15 min and flow cytometry (CytoFLEX, Beckman Coulter, :Brea, CA, United States) was used for detection.

Dog Adipose-derived Stem Cell Adipogenic/Osteogenic/Chondrogenic Differentiation Basal Medium (CAXMD-90031^1^ /CAXMD-90021^2^ /CAXMD-90041,^3^ Cyagen, China) were used to induce canine ADMSCs into adipocytes/osteocytes/chondrocytes. The differentiated cells were stained with Oil Red O, Alizarin Red, and Alcian Blue, respectively.

### Isolation of Canine Pancreatic Islets

Canine pancreatic islets were isolated by collagenase V (Sigma-Aldrich, Burlington, MA, United States) digestion and purified by centrifugation on a Ficoll density gradient (Borot et al., 2013). Purified islets were incubated (37°C, 5% CO_2_) in DMEM medium (Gibco, Waltham, MA, United States) supplemented with 10% fetal bovine serum (Zeta Life, Menlo Park, CA, United States), 100 U/mL penicillin (Sigma, Ronkonkoma, NY, United States), 0.1 mg/mL streptomycin (Sigma, Ronkonkoma, NY, United States), and 0.5 μg/mL Mycoplasma Removal Agent (MP Biomedicals, Irvine, CA, United States).

### Transdifferentiation of ADMSCs Into IPCs

**FIGURE 1:**
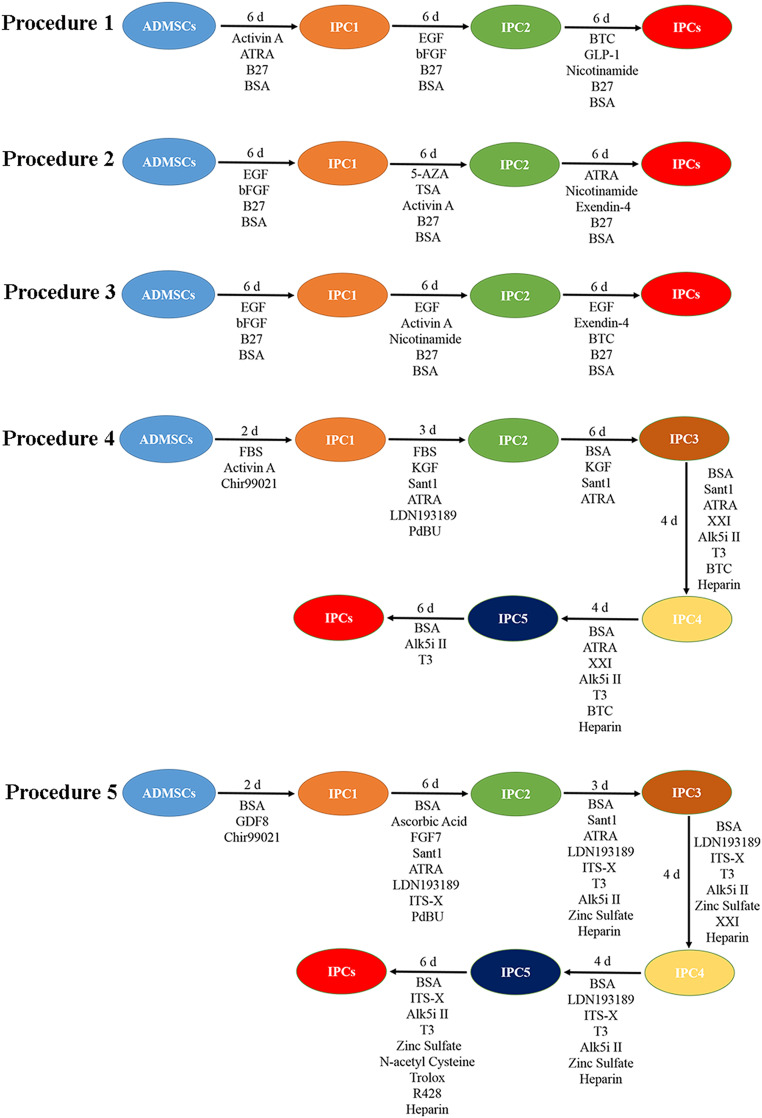
The five types of procedures.

Based on published studies, appropriate modifications to transdifferentiation procedures were developed. Finally, we used five types of procedures (Figure 1 and Supplementary Material 1) to transdifferentiate canine ADMSCs into IPCs for screening. In the initial study, the procedure 1 (Sun et al., 2007; Mohamed et al., 2016), the procedure 2 (Zhang et al., 2010), and the procedure 3 (Wang et al., 2020) can transifferentiated mesenchymal stem cells into IPCs, the procedure 4 (Pagliuca et al., 2014), and the procedure 5 (Rezania et al., 2012, 2014) can transdifferentiated induced pluripotent stem cells into IPCs. The canine ADMSCs were plated in six-well plates, and the cells were transdifferentiated when the cell density reached 75%. After transdifferentiation, cells morphology was observed, the numbers of islet-like cells were counted, and their diameters were measured. The cells were stained with dithizone using the Dithizone dyeing solution (PB9012, Coolaber, China).

### RT-qPCR

The expression of islet β-cell related genes and differentially expressed genes (DEGs) was detected by RT-qPCR in cells (Nolan et al., 2006). Canine ADMSCs were used as the control group, and *GADPH* was used as the reference gene. The TaKaRa MiniBEST Universal RNA Extraction Kit (TaKaRa, Japan) was used to extract RNA from cells. The PrimeScript^TM^ RT Master Mix (Perfect Real Time) (TaKaRa, Japan) was used to prepare cDNA. Reactions were conducted according to the Maxima SYBR Green/ROX qPCR Master Mix (ThermoFisher Scientific, Waltham, MA, United States) manual, and RT-qPCR was performed using the Step One Plus Real-Time PCR System (Applied Biosystems, Bedford, MA, United States). Three biological replicates and three technical replicates were used to determine the Ct values. The expression levels of the tested genes were determined from the Ct values, as calculated by 2^−ΔΔ*Ct*^ (Livak and Schmittgen, 2001).

### Glucose-Stimulated Insulin Secretion

Cells were washed with PBS 3 times, 5 mM glucose was added, the cells were incubated for 30 min, and the supernatant was collected. Next, cells were washed three times in PBS and incubated in 25 mM glucose for 30 min, and supernatants were collected. Finally, cells were washed three times in PBS and incubated in 5 mM glucose and 30 mM KCl for 30 min, and the supernatant was collected. Cell masses were dispersed into single cells, and cells were counted. Supernatant samples containing secreted insulin were processed using the Human/Canine/Porcine Insulin Quantikine ELISA Kit (R&D Systems, Minneapolis, MN, United States). The glucose stimulation index (SI), which measures the sensitivity of IPCs to glucose, was obtained by dividing the amount of insulin secreted at the high glucose (25 mM) level by the amount of insulin secreted at the low glucose (5 mM) level.

### Absolute Quantitative Transcriptome Sequencing Analysis

Among the five procedures, the optimal procedure was selected. Absolute Quantitative Transcriptome Sequencing Analysis (Kivioja et al., 2011) was performed on the cells obtained by the optimal transdifferentiation procedure. Cells were collected at 5 days (IPC1), 11 days (IPC2), 19 days (IPC3), 25 days (IPC4) of transdifferentiation. Canine ADMSCs were used as a negative control, and canine pancreatic islets were used as a positive control. Two biological replicates were completed for each sample.

Total RNA was extracted using TRIzol^®^ reagent (Invitrogen, Waltham, MA, United States) following the manufacturer’s protocol. mRNA was enriched, and cDNA was synthesized and connected to adapters using unique molecular identifiers (UMIs). Illumina Sequencing Platform HiSeq 2500 was used for sequencing, and the sequencing length was PE150 (Sequencing was conducted by LC Sciences, LLC.). Removal of duplicates and data error correction were performed based on genome location and UMI tagging (FastQC 0.10.1, RSeQC 2.3.9, UMI_tools 0.5.4). High quality clean reads were generated from the assembly library by filtering. HISAT2 2.0.4 (Daehwan et al., 2015) was performed to align high-quality reads with the *Canis lupus familiaris* genome,^4^ and the alignment rate was calculated. StringTie 1.3.4d (Pertea et al., 2015) was used for transcript splicing and merging. Gene expression was calculated by the fragments per kilobase per million (FPKM) method (Roberts, 2011). And edgeR (Robinson et al., 2010) was used for transcript quantification. The DEGs were selected with log2 (fold change) ≥ 2 or log2 (fold change) = −2 and the *p*-value = 0.05 by R package–edgeR (Robinson et al., 2010). If too many genes meet this *p*-value condition, use the *q*-value (the fold discovery rate *p*-value correction) = 0.05 for further screening (Flenniken and Andino, 2013). In many cases, it’s more rigorously corrected and there are fewer differential genes. The R pheatmap toolkit^5^ was used for hierarchical clustering analysis. We used GOseq (Young et al., 2010) for gene ontology (GO) enrichment analysis,^6^ Kyoto Encyclopedia of Genes, and Genomes (KEGG)^7^ for KEGG enrichment analysis in the DEGs.

### Adenovirus-Mediated Gene Overexpression

The cDNA sequences for genes were synthesized by Wuhan Gene Create Biological Engineering Co., Ltd. (Wuhan, China). The cDNA for each gene was PCR-amplified and inserted into *pAdTrack-CMV* previously digested with *BglII*/*Hin*dIII using the In-Fusion HD cloning kit (Takara, Japan) (Sleight et al., 2010). The resulting plasmid was linearized with *Pme*I, and then homologous recombination was performed with *pAdeasy-1* in *E. coli BJ5183.* The recombinant plasmid with *Pac*I linearization was used to transfect AAV-293 cells to produce recombinant adenovirus particles using the Advanced DNA RNA transfection reagent (Zeta Life, Menlo Park, CA, United States). RT-qPCR (Nolan et al., 2006) and Western-blot (following the Elabscience Western blot detection kit operation manual) were used to detect the expression of target gene. The canine ADMSCs were transfected with the recombinant adenovirus particles.

### Immunofluorescent Staining

Cells were fixed with 4% paraformaldehyde for 10 min and washed with PBS three times. Next, the cells were permeabilized for 15 min with 0.1% Triton X-100 (Sigma, Ronkonkoma, NY, United States) and washed with PBS three times. Cells were blocked with goat serum for 30 min and incubated with primary antibodies (1:600; Abcam, United Kingdom) at 4°C overnight. After washing with PBS three times, cells were incubated with secondary antibodies (1:600; Abcam, United Kingdom) for 1 h at 37°C in the dark, and again washed with PBS three times. Nucleus counterstaining was performed with 10 μg/mL Hoechst 33342 (Sigma, Ronkonkoma, NY, United States). Fluorescence images were obtained with an inverted fluorescence microscope (Sunny Optical Technology Company Limited, ICX41, China).

### siRNA-Mediated Gene Silencing

Fluorescein-labeled siRNA (Small interfering RNA) was synthesized and purified by Gene Pharma Co., Ltd. (Shanghai, China). Each gene was designed with three siRNAs. In the process of transdifferentiation, the canine ADMSCs were transfected with siRNAs using the Advanced DNA RNA transfection reagent (Zeta Life, Menlo Park, CA, United States). RT-qPCR (Nolan et al., 2006) was used to detect the silencing efficiency of siRNAs and the one with the highest silencing efficiency was selected. The gene was silenced every 7 days throughout the transdifferentiation to keep it silent.

### Statistical Analysis

Assays were repeated three times. One-way analysis of variance (ANOVA) was used for the statistical comparisons among groups. The tests were performed using IBM SPSS Statistics 25 software (SPSS Inc., Chicago, IL, United States).

## Results

### Canine ADMSC Isolation, Culture, and Identification

The fourth-generation isolated cells showed long spindle type and adherent growth (Supplementary Figure 2A) and proliferated rapidly in the 3–7 day after adherent growth, exhibiting logarithmic growth (Supplementary Figure 2B). The cells were tested by flow cytometry, and the expression levels of CD13, CD29, CD44, CD73, CD90, and CD105 were positive. Expression levels for CD31, CD45, and CD235a were negative (Supplementary Figure 2C). In osteogenic differentiation, the cells grew in clusters; Alizarin Red staining showed red-stained calcified nodules. In chondrogenic differentiation, the cells gathered and grew, and blue staining was observed after Alcian Blue staining. In adipogenic differentiation, large areas of fat droplets were observed by Oil Red O staining (Supplementary Figure 2D). These results prove that the isolated cells were canine ADMSCs.

### Transdifferentiation of ADMSCs Into IPCs

**FIGURE 2:**
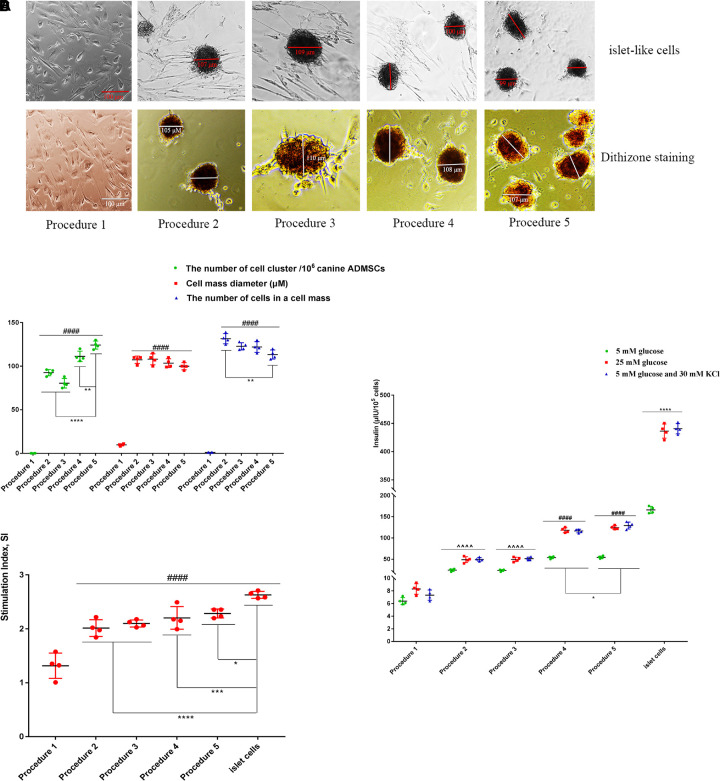
The transdifferentiation of ADMSCs into IPCs using five types of procedures. **(A)** The cellular morphology and dithizone staining. **(B)** The number of cell cluster of procedures 2, 3, 4, 5 was significantly more than inducing procedures 1 (####*p* < 0.0001). There were also differences in the number of cell cluster in procedures 2, 3, 4, and 5 (****p < 0.0001; **p < 0.01) (*n* = 4). **(C)** After the stimulation of low glucose (5 mM), high glucose (25 mM), 5 mM glucose, and 30 mM KCl, the secretion of insulin in procedure 1 was significantly lower than that of procedures 2 and 3 (^^^^*p* < 0.0001). The secretion of insulin in procedure 4 and 5 was higher than that of procedures 2 and 3 (####*p* < 0.0001), procedure 5 is higher than procedure 4 (*p < 0.05), but they were all lower than the mature islet cells group (****p < 0.0001) (*n* = 4). **(D)** The glucose Stimulation Index (SI) of procedure 1 was significantly lower than that of procedures 2, 3, 4, 5, and mature islet cells (####*p* < 0.0001), procedure 5 is highest in five types of procedures (****p < 0.0001; ***p < 0.001; *p < 0.05) (*n* = 4).

The canine ADMSCs were transdifferentiated into IPCs using five types of procedures. In procedure 1, the cells did not form into clusters, and no obvious islet-like cells were found. In procedures 2, 3, 4, and 5, the cells agglomerated into a spherical shape, with obvious islet-like cells; the resulting islet-like cells were scarlet with dithizone staining (Figure 2A). There were also differences in the number of cell cluster from procedures 2, 3, 4, and 5, among which the number of procedure 5 cells was the greatest, reaching 123 ± 10.72/10^6^ canine ADMCS, with a cell cluster of 99.75 ± 8.26 μm in diameter and an average of 110.50 ± 18.81 cells per cell cluster (Figure 2B). For procedure 1, after stimulation with low glucose (5 mM), high glucose (25 mM), 5 mM glucose, and 30 mM KCl, the secretion of insulin was significantly lower than values for procedures 2, 3, 4, and 5. The secretion of insulin was the highest in procedure 5, with 53.13 ± 3.39 IU/10^5^ cells in low glucose, 125.23 ± 4.35 IU/10^5^ cells in high glucose and 127.02 ± 4.66 IU/10^5^ cells in 5 mM glucose and 30 mM KCl; the second highest level of insulin secretion was observed for procedure 4, but all these values lower than for the mature islet cells group (Figure 2C). The glucose SI after procedure 1 was significantly lower than values for procedures 2, 3, 4, and 5; the SI of procedure 5 was 2.36 ± 0.11, showing the most favorable response to glucose stimulation (Figure 2D).

**FIGURE 3:**
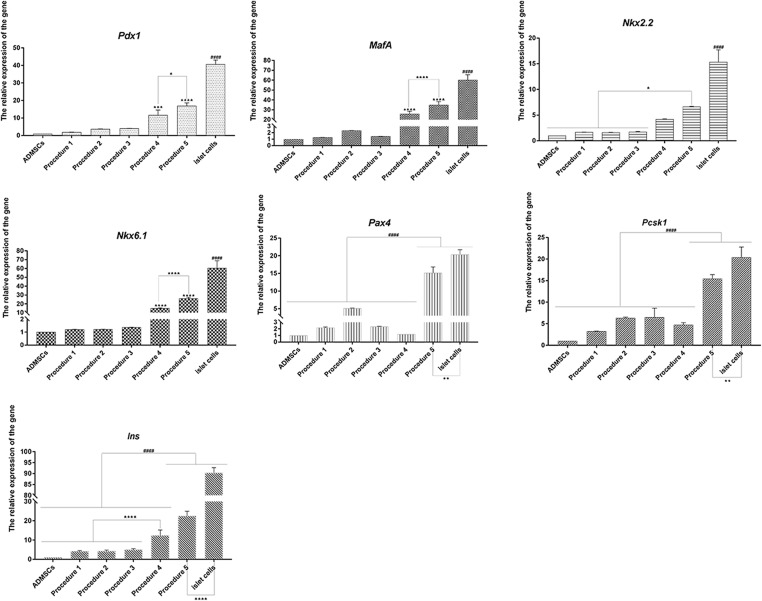
RT-qPCR analysis. The expression level of each gene in procedure 5 was elevated, which was significantly higher than procedures 1, 2, 3, and 4, there is no difference between procedures 1, 2, and 3. The expressions of *Pdx1*, *MafA*, *Nkx6.1*, and *Ins* in procedure 4 were significantly higher than those in procedure 1, 2, and 3 (****p < 0.0001; ***p < 0.001; **p < 0.01; *p < 0.05). The expression level of all genes in procedure 1, 2, 3, 4, and 5 was significantly lower than that of mature islet cells (####*p* < 0.0001).

The RT-qPCR was performed on the cells (the primers are provided in Supplementary Table 1 at Supplementary Material 3). As shown in Figure 3, the expression level of each gene (Canine ADMSCs were used as the control group, and *GADPH* was used as the reference gene, the relative expression of the genes calculated by 2−^ΔΔ*Ct*^) was elevated following procedure 5 and significantly different from values for procedures 1, 2, 3, and 4. The expression levels for procedures 1, 2, 3, 4, and 5 were significantly lower than those in mature islet cells.

According to the above results, procedure 5 exhibited the highest transdifferentiation efficiency.

### Quality Control of Sequencing Data and Genes Expression Level Analysis

Absolute Quantitative Transcriptome Sequencing Analysis was performed for the four stages of procedure 5 (divided into IPC1, IPC2, IPC3, and IPC4). Canine ADMSCs were used as a negative control (divided into ADSC) and mature canine islet cells as a positive control (divided into Beta_cell). After sequencing, the raw reads were saved in FASTQ format. Supplementary Table 1 in Supplementary Material 4 lists the data quality throughout the data analysis process. After data processing, highly reliable data were aligned to the canine reference genome to obtain comprehensive transcript information (Supplementary Table 2 in Supplementary Material 4). The regional distribution aligned with the reference genome is shown in Supplementary Material 5. The result of genes expression level analysis (Transcriptome expression profile and Gene expression profiling) was shown in Supplementary Material 6, a total of 60,151 transcripts were obtained.

The problem of oncological transformation of stem cells is acute in the development of molecular stem cell technologies. In this study, tumor markers, such as C*d133*, *A2b5*, *Ssea-1* were not expressed, and *Myc* had low expression in ADSC, IPC1, IPC2, IPC3, IPC4, and Beta_cell.

### Differential Expression Genes Analysis and Clustering Analysis

**FIGURE 4:**
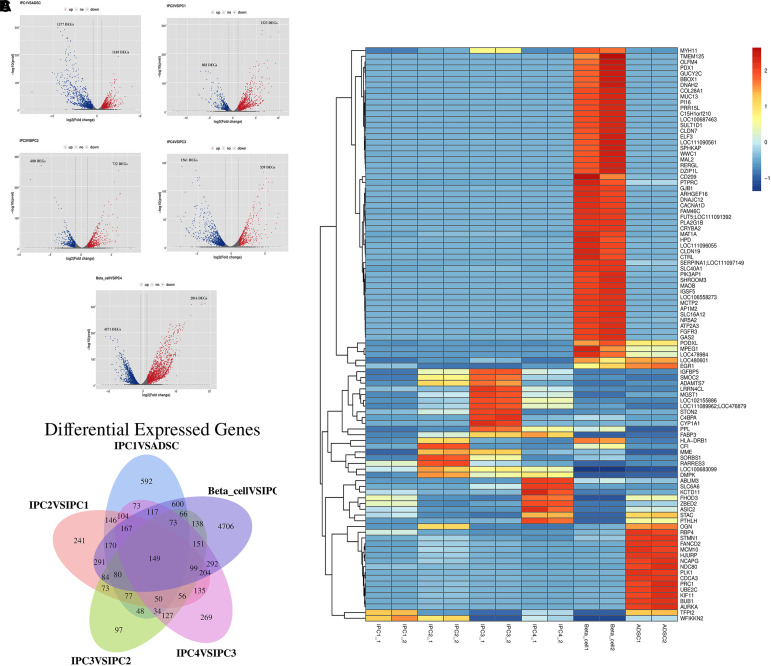
Differential expression genes analysis and clustering analysis. **(A)** Volcanic maps of the DEGs. Blue dot and green dot represent that the expression level of genes has difference, the black dot represents no difference. **(B)** Clustering analysis. The abscissa is the sample, and the ordinate is the genes. Different colors indicate different gene expression level, and the color ranges from blue to white to red, indicating low to high expression levels. **(C)** Venn diagrams was constructed to compare the spectra of DEGs with each other.

The DEGs screening conditions for the DEGs were log2 (fold change) ≥ 2 or log2 (fold change) = −2 with the *p*-value set at = 0.05. The fold discovery rate *p*-value correction (FDR, *q*-value) is also provided in the results. DEGs were divided into five groups: IPC1 vs. ADSC (1169 up-regulated genes and 1377 down-regulated genes, *p*-value = 0.05; 1166 up-regulated genes and 1376 down-regulated genes, *q*-value = 0.05), IPC2 vs. IPC1 (1323 up-regulated genes and 803 down-regulated genes, *p*-value = 0.05; 1319 up-regulated genes and 799 down-regulated genes, *q*-value = 0.05), IPC3 vs. IPC2 (722 up-regulated genes and 680 down-regulated genes, *p*-value = 0.05; 721 up-regulated genes and 678 down-regulated genes, *q*-value = 0.05), IPC4 vs. IPC3 (539 up-regulated genes and 1561 down-regulated genes, *p*-value = 0.05; 537 up-regulated genes and 1525 down-regulated genes, *q*-value = 0.05), and Beta_cell vs. IPC4 (2816 up-regulated genes and 4571 down-regulated genes, *p*-value = 0.05; 2815 up-regulated genes and 4472 down-regulated genes, *q*-value = 0.05) (Supplementary Material 7). The volcanic diagram of each differentially expressed gene analysis group is shown in Figure 4A. After FPKM values of DEGs were transformed to *Z* values, a clustering diagram was drawn (Figure 4B). Venn diagrams was constructed to compare the spectra of DEGs with each other (Figure 4C).

*Pdx1* (*p*-value = 0.00, *q*-value = 0.00), *Nkx6.1* (*p*-value = 0.00, *q*-value = 0.00), *Nkx2.2* (*p*-value = 0.00, *q*-value = 0.00), *Pax4* (*p*-value = 0.00, *q*-value = 0.00), and *Ins* (*p*-value = 0.00, *q*-value = 0.00), which are critical for islet β-cell development and insulin secretion, were also screened for DEGs; *MafA* was not differentially expressed in this study (Supplementary Material 7). DEGs analysis and cluster analysis showed obvious differences in cell genes expression appears from canine ADMSCs to IPC4, and there were significant differences between IPC4 and islet cells. These results suggest that at the end of each transdifferentiation phase, the cells gene expression undergo corresponding changes.

### GO Functional Enrichment Analysis and KEGG Pathways Enrichment Analysis

**FIGURE 5:**
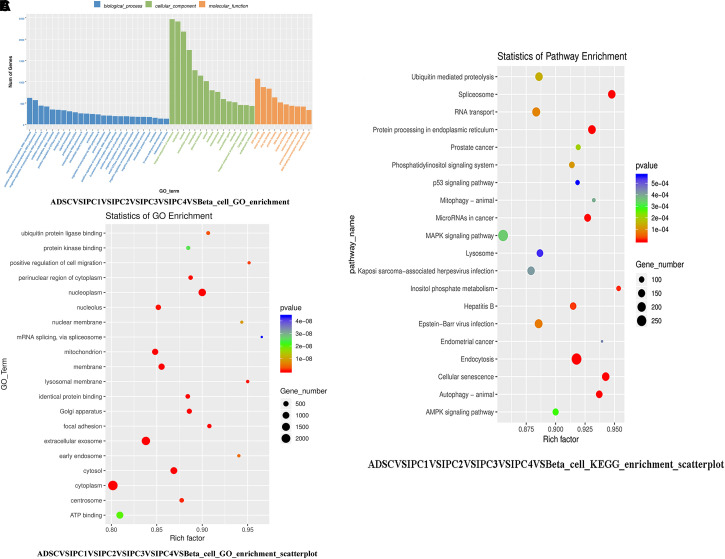
The GO functional enrichment analysis and KEGG pathways enrichment analysis. **(A)** The GO enrichment analysis of all DEGs. **(B)** The 20 most significant GO terms in GO functional enrichment analysis of DEGs. The horizontal axis represents the enrichment degree (Rich factor), the vertical axis represents the GO Term of enrichment. Dot size indicates the number of DEGs enriched in a GO term. The dot color represents different *P*-values; Rich factor means the number of DEGs belonging to a certain GO term. The higher the Rich factor is, the higher the GO enrichment degree will be. **(C)** The 20 most significant KEGG pathways in KEGG Pathways Enrichment Analysis of DEGs.

Supplementary Material 8 and Figure 5A show the GO enrichment analysis for all DEGs. The fold discovery rate *p*-value correction (FDR, *q*-value) is also provided in the results. There were 47 GO Terms enriched by over 100 DEGs (*p*-value = 0.05 and *q*-value = 0.05), 26 GO Terms enriched by over 200 DEGs (*p*-value = 0.05 and *q*-value = 0.05). Supplementary Material 9A shows the GO enrichment analysis of each group. According to the size of *p*-values and *q*-values, the 20 most significant GO terms were selected as dot plots (Figure 5B), Supplementary Material 9B shows the 20 most significant GO terms for each group.

In GO Functional Enrichment Analysis, we identified 126 DEGs involved in endocrine pancreas development (*p*-value = 0.34, *q*-value = 0.93), pancreas morphogenesis (*p*-value = 0.42, *q*-value = 0.93), pancreas development (*p*-value = 0.45, *q*-value = 0.93), branching involved in pancreas morphogenesis (*p*-value = 0.42, *q*-value = 0.93), regulation of insulin secretion involved in cellular response to glucose stimulus (*p*-value = 0.02, *q*-value = 0.73), insulin secretion (*p*-value = 0.15, *q*-value = 0.93), positive regulation of insulin secretion involved in cellular response to glucose stimulus (*p*-value = 0.16, *q*-value = 0.93), insulin secretion involved in cellular response to glucose stimulus (*p*-value = 0.17, *q*-value = 0.93), positive regulation of insulin secretion (*p*-value = 0.16, *q*-value = 0.93), regulation of insulin secretion (*p*-value = 0.33, *q*-value = 0.93). In IPC1VSADSC, there are 18 DEGs were enriched in these GO Terms, In IPC2VSIPC1, IPC3VSIPC2, IPC4VSIPC3, and Beta_cellVSIPC4, there were 15 DEGs, 14 DEGs, 19 DEGs, and 90 DEGs were enriched in these GO Terms, respectively. See Supplementary Material 10 for specific DEGs names, these DEGs are involved in pancreatic islet cell development, insulin secretion. These DEGs may play important roles in the transdifferentiation of canine ADMSCs into IPCs *in vitro*.

In this study, scatterplots were used to visually demonstrate KEGG pathway enrichment results of DEGs. The 20 KEGG pathways with the most significant expression were selected according to *p*-values and *q*-values (Figure 5C). Supplementary Material 11 shows the 20 most significant KEGG pathways for each group. There were 23 KEGG Pathways enriched by over 100 DEGs (*p*-value = 0.05 and *q*-value = 0.05), 3 KEGG Pathways enriched by over 200 DEGs (*p*-value = 0.05 and *q*-value = 0.05) (Supplementary Material 12).

In KEGG Pathways Enrichment Analysis, we found that there were 143 DEGs, 74 DEGs, 23 DEGs, and 26 DEGs enriched, respectively, for Insulin signaling pathway (*p*-value = 0.01, *q*-value = 0.06), Insulin secretion (*p*-value = 0.42, *q*-value = 0.80), Maturity onset diabetes of the young (*p*-value = 0.15, *q*-value = 0.39), and Type I diabetes mellitus (*p*-value = 0.99, *q*-value = 1.00). In IPC1VSADSC, there are 31 DEGs were enriched in these KEGG Pathways, In IPC2VSIPC1, IPC3VSIPC2, IPC4VSIPC3, and Beta_cellVSIPC4, there were 49 DEGs, 28 DEGs, 39 DEGs, and 178 DEGs were enriched in these KEGG Pathways, respectively (see Supplementary Material 12 for specific DEGs names). These DEGs can regulate the development of pancreatic islet cells and the production of insulin. These DEGs may be important for transdifferentiation of canine ADMSCs into IPCs *in vitro*.

*Pdx1*, *Nkx6.1*, *Nkx2.2*, *MafA*, and *Pax4* have been shown to play an important role in the transdifferentiation of mesenchymal stem cells to IPCs (Limbert et al., 2011; Bahrebar et al., 2015; Li et al., 2017; Zhang et al., 2019; Aigha and Abdelalim, 2020). In this study, the aim was to explore what other factors, except for these key regulatory factors, play a role in the transdifferentiation of mesenchymal stem cells to IPCs. So we decided to filter out these genes and look for new regulatory genes instead. In this study, gene functional analysis was performed on the genes involved in the development of pancreatic islet β-cells, DEGs listed from GO functional enrichment analysis and KEGG pathways enrichment analysis, we screened a total of 51 genes according to the significance of difference, the expression level at each stage and the function of genes (Supplementary Material 13). Protein interaction network analysis was performed (Supplementary Material 14), we found that FOXO1, FOXA1, SOX17, HNF1A, HNF1B, INSM1, NR5A2, GCK, PBX1, ONECUT1, ONECUT2, PTF1A, and RFX6 can form a protein interaction network with PDX1, NKX6.1, NKX2.2, MAFA, NGN3, and PAX4. Although WNT5A, TCF7L2, BMP4, RFX3, and DLL1 cannot interact with PDX1, NKX6.1, NKX2.2, MAFA, NGN3, and PAX4, it does interact with FOXA1, SOX17, NR5A2, ONECUT1, HNF1A, HNF1B, and GCK, that is, it indirectly interacts with PDX1, NKX6.1, NKX2.2, MAFA, NGN3, and PAX4. The 18 genes (*Foxo1*, *Foxa1*, *Sox17*, *Hnf1a*, *Hnf1b*, *Insm1*, *Nr5a2*, *Gck*, *Pbx1*, *Onecut1*, *Onecut2*, *Ptf1a*, *Wnt5a*, *Tcf7l2*, *Bmp4*, *Rfx3*, *Dll1*, *and Rfx6*) are referred to as novel functional genes. The 18 novel functional genes were separately overexpressed in canine ADMSCs. Novel functional genes were further screened by RT-qPCR validation (Canine ADMSCs were used as the control group, and GADPH was used as the reference gene, the relative expression of the genes calculated by 2^-△△Ct). Relative to other genes, the *Foxa1*, *Hnf1b*, *Dll1*, *Pbx1*, *and Rfx3* can significantly stimulate the expression of islet β-cell development cascade regulation genes (Supplementary Material 15). The *Foxa1*, *Hnf1b*, *Dll1*, *Pbx1*, and *Rfx3* were used in subsequent studies.

### RT-qPCR Verification of Absolute Quantitative Transcriptome Sequencing

In this study, 12 DEGs were selected from the above DEGs, and the Absolute Quantitative Transcriptome Sequencing Analysis results were verified by RT-qPCR (primers listed in Supplementary Table 2 at Supplementary Material 3). In Absolute Quantitative Transcriptome Sequencing, we used the PRKM results (Supplementary Material 6). In RT-qPCR, we used the relative expression of the genes (Canine ADMSCs were used as the control group, and *GADPH* was used as the reference gene, calculated by 2^–Δ^ ^Δ^ ^*Ct*^) (Supplementary Material 16). The results of RT-qPCR were consistent with the gene expression trend in the Absolute Quantitative Transcriptome Sequencing, and the Absolute Quantitative Transcriptome Sequencing results were correct.

### Preparation of Adenovirus Venom

*Foxa1*, *Hnf1b*, *Dll1*, *Pbx1*, and *Rfx3* overexpression adenovirus particles were prepared to verify the roles of these five genes in the differentiation of canine ADMSCs into IPCs. Five genes were amplified by PCR (primers listed in Supplementary Table 3 in Supplementary Material 3), and five overexpressed single-gene adenovirus shuttle vectors were constructed (*pADTrcak-CMV-Foxa1*, *pADTrcak-CMV-Hnf1b*, *pADTrcak-CMV-Dll1*, *pADTrcak-CMV-Pbx1*, and *pADTrcak-CMV-Rfx3*). And the recombinant vectors were *pAdeasy-Foxa1*, *pAdeasy-Hnf1b*, *pAdeasy-Dll1*, *pAdeasy-Pbx1*, and *pAdeasy-Rfx3*. The linearized recombinant vectors were packaged as adenoviruses by AAV-293 cells, and the adenovirus particles were obtained; the virus titers were 6 × 10^9^, 5.5 × 10^9^, 7.5 × 10^9^, 6 × 10^9^, and 7 × 10^9^ TU/mL, respectively. The expression of the target genes and proteins was verified by RT-qPCR (primers listed in Supplementary Table 4 in Supplementary Material 4) and Western-blot (Supplementary Material 17). The results showed that the adenovirus particles could successfully express the target proteins.

### Functional Verification of the *Foxa1*, *Hnf1b*, *Dll1*, *Pbx1*, and *Rfx3*

The canine ADMSCs were infected with the adenovirus particles according to the multiplicity of infection MOI = 100. Two days after canine ADMSCs were infected, positive cells were screened according to green fluorescence. The cells were cultured for 2 days and transdifferentiated with procedure 5 (grouped into FOXA1 + Procedure 5, HNF1B + Procedure 5, DLL1 + Procedure 5, PBX1 + Procedure 5, RFX3 + Procedure 5) with normal cell passage during transdifferentiation. After 25 days of transdifferentiation, the cells were observed.

**FIGURE 6:**
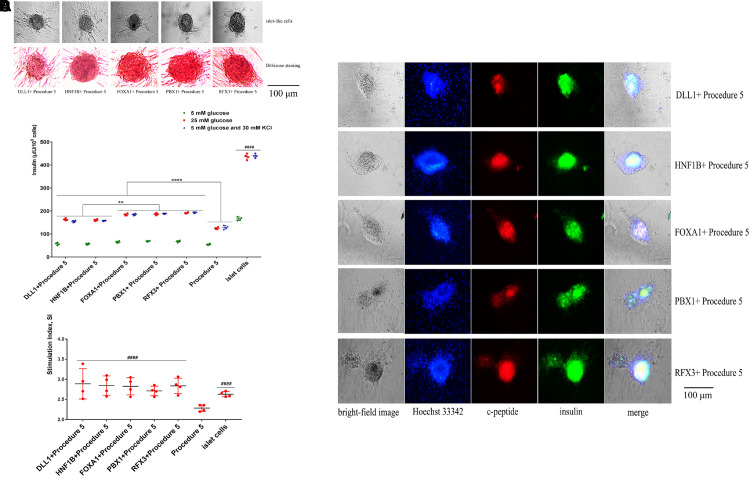
Functional verification of the *Foxa1*, *Hnf1b*, *Dll1*, *Pbx1*, and *Rfx3*. **(A)** The cells agglomerated into a spherical shape, with obvious islet-like cells. The cells were stained with dithizone, and the cells could be dyed red. **(B)** After the stimulation of low glucose (5 mM), high glucose (25 mM), 5 mM glucose, and 30 mM KCl, the secretion of insulin in procedure 5 was significantly lower than that of genes overexpression groups (****p < 0.0001). The secretion of insulin in FOXA1 + Procedure 5, PBX1 + Procedure 5, and RFX3 + Procedure 5 was highest (**p < 0.01), but they were all lower than the mature islet cells group (####*p* < 0.0001) (*n* = 4). **(C)** The glucose stimulation index of genes overexpression is greater than 2.5, and higher than procedure 5 (####*p* < 0.0001) (*n* = 4). **(D)** Immunofluorescence staining of insulin and c-peptide showed that the cells were insulin and c-peptide positive. Green was insulin, red was c-peptide, and Hoechst 33342 made the nucleus blue.

**FIGURE 7:**
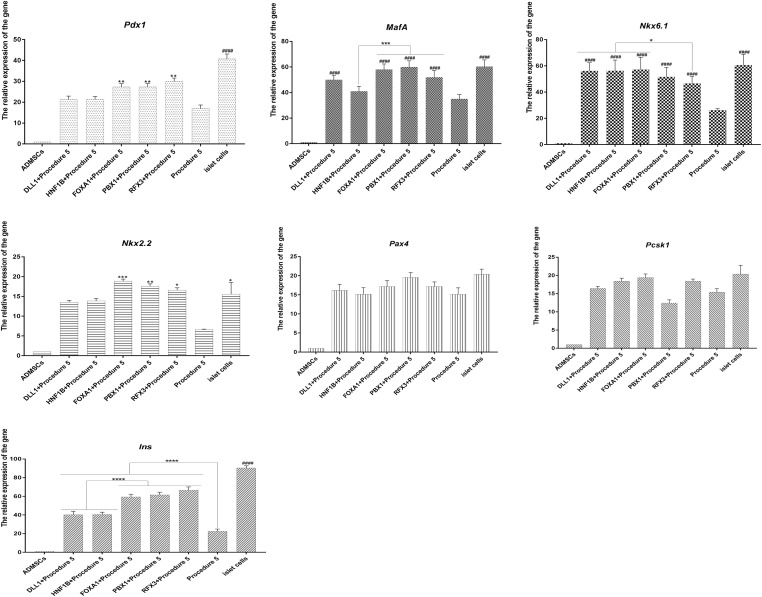
RT-qPCR analysis. The overexpression of *Foxa1*, *Hnf1b*, *Dll1*, *Pbx1*, *Rfx3* can significantly stimulate high expression of islet cascade regulatory genes, with higher expression level than procedure 5. After *Foxa1*, *Pbx1*, and *Rfx3* were overexpressed, the expression of *Pdx1*, *Ins*, and *Nkx2.2* was significantly higher than that of the unexpressed group and *Hnf1b and Dll1* overexpressed groups. There was no significant difference in the expression of *Pax4* and *Pcsk1* (####*p* < 0.0001; ****p < 0.0001; ***p < 0.001; **p < 0.01; *p < 0.05).

The cells of five groups grew in clusters, showing the appearance of islets. The green fluorescence carried by adenoviruses had disappeared, the cells were stained with dithizone and were able to be dyed red (Figure 6A), indicating that the cells could express insulin. The cells of five groups under the stimulus of glucose were able to secrete insulin, and insulin secretion was higher than procedure 5 (Figure 6B). The overexpression of *Foxa1*, *Hnf1b*, *Dll1*, *Pbx1*, *Rfx3* were further able to improve the effect of transdifferentiation, insulin secretion. Among these, the overexpression of *Foxa1*, *Pbx1*, and *Rfx3* exhibited the most significant effects. The glucose SI exceeded 2.5 for five groups, higher than for procedure 5, indicating that cells were able to respond to glucose stimulation (Figure 6C). Immunofluorescence staining of insulin and c-peptide showed that the cells were insulin and c-peptide positive (Figure 6D). The expression of islet-specific transcription factors was detected by RT-qPCR (primers listed in Supplementary Table 1 in Supplementary Material 3); the overexpression of *Foxa1*, *Hnf1b*, *Dll1*, *Pbx1*, *Rfx3* were able to significantly stimulate high expression of *Pdx1*, *MafA*, *Nkx6.1*, *Nkx2.2*, and *Ins* genes with higher levels of expression than for procedure 5, indicating that the overexpression of these five genes could further stimulate the expression of islet cascade regulatory genes and improve transdifferentiation efficiency (Canine ADMSCs were used as the control group, and *GADPH* was used as the reference gene, the relative expression of the genes calculated by 2^-△△Ct) (Figure 7). The results described above indicate that the overexpression of *Foxa1*, *Hnf1b*, *Dll1*, *Pbx1*, and *Rfx3* was able to increase transdifferentiation efficiency and improve maturity of IPCs; *Foxa1*, *Pbx1*, and *Rfx3* exerted the most significant effects.

### siRNA-Mediated Genes Silencing

Each gene was designed with three siRNAs (Supplementary Table 5 in Supplementary Material 3), and the one with the highest silencing efficiency was selected. The canine ADMSCs were transfected with the siRNAs every 7 days. After transfection, cells were induced with procedure 5 (grouped into: FOXA1 SiRNA + Procedure 5, HNF1B SiRNA + Procedure 5, DLL1 SiRNA + Procedure 5, PBX1 SiRNA + Procedure 5, RFX3 SiRNA + Procedure 5).

**FIGURE 8:**
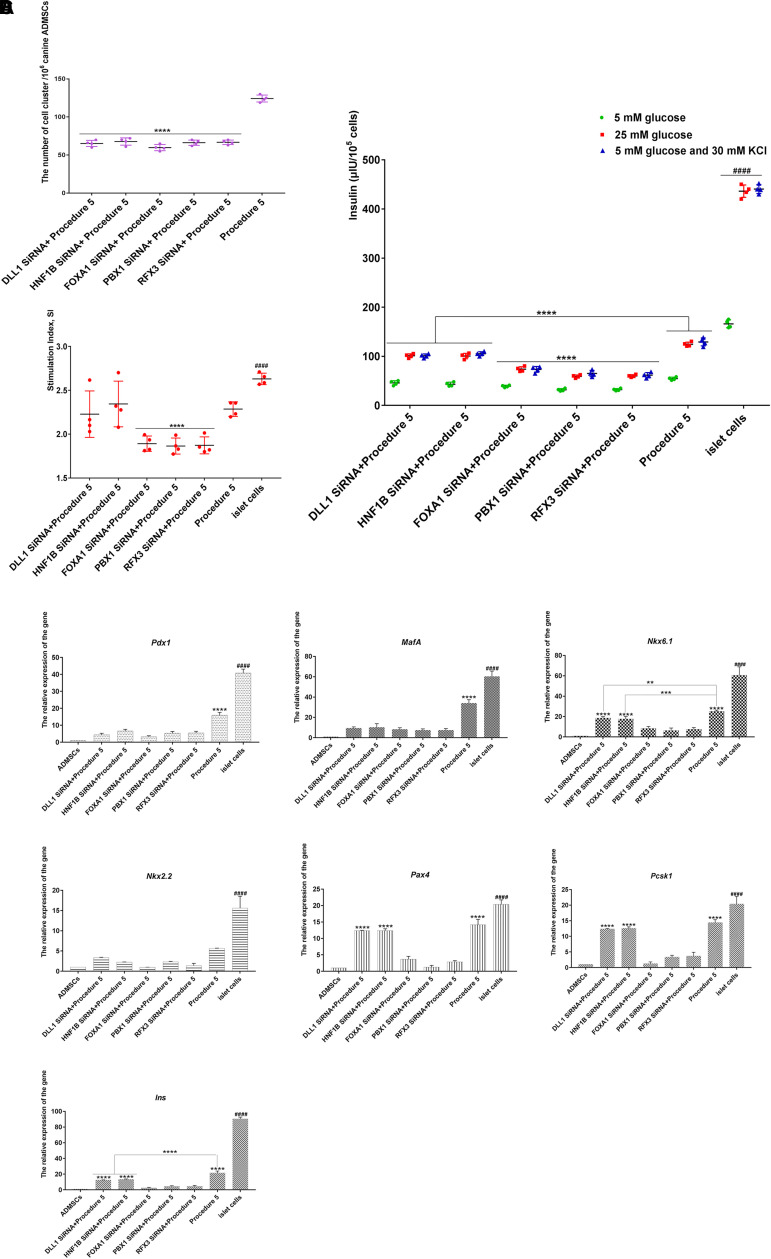
SiRNA-mediated genes silencing. **(A)** After SiRNA infects canine ADMSCs, the numbers of cluster in gene silencing groups were significantly less than that of the non-silenced group (****p < 0.0001) (*n* = 4). **(B)** After the stimulation of low glucose (5 mM), high glucose (25 mM), 5 mM glucose, and 30 mM KCl, the secretion of insulin in procedure 5 was significantly higher than that of genes silence groups (****p < 0.0001). The secretion of insulin in FOXA1 SiRNA + Procedure 5, PBX1 SiRNA + Procedure 5, and RFX3 SiRNA + Procedure 5 was lowest (****p<0.0001), they were all lower than the mature islet cells group (####*p* < 0.0001) (*n* = 4). **(C)** The glucose stimulation index of FOXA1 SiRNA + Procedure 5, PBX1 SiRNA + Procedure 5, and RFX3 SiRNA + Procedure 5 was lower than procedure 5, DLL1 SiRNA + Procedure 5, HNF1B SiRNA + Procedure 5 (****p < 0.0001), islet cells group was highest (####*p* < 0.01) (*n* = 4). **(D)** The silence of *Foxa1*, *Hnf1b*, *Dll1*, *Pbx1*, *Rfx3* can reduce expression of islet cascade regulatory genes. Islet cascade regulatory genes were most significantly decreased when *Foxa1*, *Pbx1*, *Rfx3* genes were silenced separately compared with the non-silenced group. After *Foxa1*, *Pbx1*, and *Rfx3* were silenced, the expression of *Nkx6.1*, *Pax4*, *Ins*, and *Pcsk1* was significantly lower than that of the *Hnf1b and Dll1* silencing groups. There was no significant difference in the expression of *Nkx2.2* (****p < 0.0001; ***p < 0.001; **p < 0.01). The expression of all genes was lower than the mature islet cells group (####*p* < 0.0001).

After *Foxa1*, *Hnf1b*, *Dll1*, *Pbx1*, and *Rfx3* were separately silenced, the cells showed aggregation, but numbers of clusters were significantly less than those of the non-silenced groups (Figure 8A). When the cells were subjected to the glucose stimulation test, it was found that the ability of the cells to secrete insulin decreased compared with the non-silenced groups; secretion declined significantly with FOXA1 siRNA, PBX1 siRNA, and RFX3 siRNA, indicating that the transdifferentiation efficiency of the cells was reduced after genes silencing (Figure 8B). Decreases in the glucose SI indicated reduced sensitivity to glucose with FOXA1 siRNA, PBX1 siRNA, and RFX3 siRNA (Figure 8C). The expression of islet-specific transcription factors was detected by RT-qPCR (Canine ADMSCs were used as the control group, and *GADPH* was used as the reference gene, the relative expression of the genes calculated by 2^-△△Ct) (primers listed in Supplementary Table 1 in Supplementary Material 2), and it was found that the expression of *Pdx1*, *MafA*, *Nkx6.1*, *Nkx2.2*, *Pax4*, *Pcsk1*, and *Ins* genes was reduced when *Foxa1*, *Hnf1b*, *Dll1*, *Pbx1*, and *Rfx3* genes were separately silenced compared with the non-silenced groups. FOXA1 siRNA, PBX1 siRNA, and RFX3 siRNA decreased most significantly (Figure 8D). These results indicate that *Foxa1*, *Hnf1b*, *Dll1*, *Pbx1*, and *Rfx3* were silenced, and the transdifferentiation efficiency and IPC maturity were depressed, the transdifferentiation efficiency most significantly decreasing after the silencing of *Foxa1*, *Pbx1*, and *Rfx3*.

## Discussion

### Transdifferentiation of Canine ADMSCs Into IPCs

Retinoic acid and fibroblast growth factor are essential for pancreatic development, at present, most procedures include agonists for these signaling pathways (Bhushan et al., 2001; Molotkov et al., 2005). However, bone morphogenetic protein (BMP) signaling has been shown to promote choice of liver destiny rather than pancreas development (Wandzioch and Zaret, 2009). Accordingly, several procedures involve BMP inhibitors. However, it has also been suggested that BMP inhibitors should be eliminated because they have been shown to promote premature endocrine differentiation and damage PDX1/NKX6.1 positive cells (Russ et al., 2015). Studies have shown that histone deacetylase inhibitors could significantly improve the morphological grading and insulin secretion of islet cells (Ikemoto et al., 2018). There has also been no consensus on whether other pathway regulators, such as epidermal growth factor (EGF) or protein kinase C (PKC) agonists, should be included in procedures (Rezania et al., 2014; Nostro et al., 2015; Russ et al., 2015).

In this study, we systematically compared five procedures, each of which used different regulators, including agonists and inhibitors of various signaling pathways, transdifferentiation steps, and transdifferentiation times. In procedure 1, cells unable to respond to glucose stimulation, and no islet-like cells appeared in procedure 1. In procedures 2 and 3, insulin secretion and glucose SI were significantly higher than for procedure 1. Procedures 1, 2, and 3 need to be improved to further improve insulin secretion and cell maturity. Insulin secretion and glucose SI were highest in procedures 4 and 5, procedure 5 is higher than procedure 4. In inducing procedures 2, 3, 4, and 5, insulin secretion increased with increasing formation of islet-like cells, this suggests that cell formation contributes to the maturation of cells. With respect to cell cluster diameter and the number of cells that a cell cluster contains, a bigger cell cluster is not more favorable. The results in this study showed that the transdifferentiation effect improved as the cell number and diameter of the cell cluster decreased, showing greater insulin secretion, but the optimal cell cluster diameter and number; i.e., the threshold values, are unknown and require further study. In the original study, procedure 1, 2, and 3 can transdifferentiate mesenchymal stem cells into IPCs, and procedure 4, and 5 can transdifferentiate pluripotent stem cells into IPCs. However, in this study, the transdifferentiation efficiency of procedures 4 and 5 was higher than that of procedures 1, 2, and 3, indicating that further modification of procedures 1, 2, and 3 was required, and that the transdifferentiation procedure suitable for induced pluripotent stem cells was also suitable for mesenchymal stem cells, and better efficiency could be obtained. Although procedure 5 achieved a good induction efficiency, it still lagged far behind the insulin secretion capacity of mature islet β-cells, which was caused by the limitations of *in vitro* culture conditions. No matter how perfect the *in vitro* conditions were, they could not be completely the same as the complex *in vivo* development environment. In addition, the transdifferentiation procedures in this study was carried out in two-dimensional mode, which had a certain impact on the efficiency of transdifferentiation. In 2019, Mohammad Foad Abazari et al., found that the expression levels of *Ins*, *Glut2*, and *Pdx1* genes in cells induced by three-dimensional culture were significantly higher than those in cells cultured by two-dimensional culture (Abazari et al., 2020). In the following studies, we will conduct transdifferentiation in three-dimensional mode to explore the changes in genes and metabolic pathways during the transdifferentiation of ADMSCs to IPCs in three-dimensional mode.

In this study, the optimal transdifferentiation procedure (procedure 5) was determined through comparison with various detection methods, and this laid a good foundation for quantitative Absolute Quantitative Transcriptome Sequencing to generate better genetic datasets and identify novel functional genes.

### Absolute Quantitative Transcriptome Sequencing in Transdifferentiation of Canine ADMSCs Into IPCs

How is the transdifferentiation of ADMSCs into islet cells different from the maturation process of natural islet cells? What new transcription factors are involved in regulation besides Pdx1, Nkx6.1, Ngn3, and other transcription factors that are known to play an important role.

In 2019, a study evaluated three transdifferentiation procedures, sequenced the resulting pancreatic progenitor cells with mRNA and ATAC, and compared them with a human embryonic pancreatic population. This study defined a common transcriptional and epigenetic signature of PPs, including several genes not previously involved in pancreatic development (Wesolowska-Andersen et al., 2020). In 2020, Wang et al., completed the transdifferentiation of BMSCs into IPCs process and achieved the transcriptome profiling of five samples with two biological duplicates. A total of 11,530 DEGs were revealed in the profiling data. In KEGG enrichment analysis, DEGs are mainly concentrated in tight junction, protein digestion and absorption, pancreatic secretion, focal adhesion, ECM-receptor interaction, Rap1 signaling pathway, and cell cycle, etc. In GO enrichment analysis, DEGs are mainly concentrated in the categories of nucleus, extracellular region, intracellular membrane-bound organelle, the regulation of transcription, regulation of RNA biosynthetic process, carbohydrate metabolic process, single-organism carbohydrate metabolic process and small GTPase-mediated signal transduction, et al. *Sstr2*, *Rps6ka6*, and *Vip* they pick up may regulate decisive genes during the development of transdifferentiation of insulin producing cells (Wang et al., 2021). In this study, Absolute Quantitative Transcriptome Sequencing was used to detect the changes of genes and metabolic pathways during the transdifferentiation of canine ADMSCs into IPCs *in vitro* for the first time. In this sequencing, we obtained a large genetic database, which provided a certain reference for the study of ADMSCs transdifferentiating into IPCs, islet development, and canine gene pool. A total of 15,561 DEGs were revealed in the profiling data, 4031 more DEGs were found than the study by Wang et al. The *Sstr2*, *Rps6ka6*, and *Vip* selected by Wang et al., showed no specificity in this study and were not selected. In GO and KEGG enrichment analysis, the signal pathways and functions enriched by DEGs were also different compared with those studied by Wang et al. Only a few signal pathways were the same. The transcriptome data of the two studies are partly the same, but also partly different. The main reasons are as follows: The first is the comparison between BMSCs and ADMSCs, the second is the application of Absolute Quantitative Transcriptome Sequencing technology, in Absolute Quantitative Transcriptome Sequencing, UMI technology is used to tag each sequence segment to eliminate interference with the quantitative accuracy of the transcriptome by PCR amplification to the maximum extent and to obtain more accurate quantitative analysis results (Kivioja et al., 2011; Islam et al., 2014). And the third is the difference of transdifferentiation procedures. The transdifferentiation procedures (the procedure 3 in this study) used in the study of Wang et al., were found in this study to be of low transdifferentiation efficiency, but this study compared the transdifferentiation procedures. The optimal procedure (procedure 5) was selected for Absolute Quantitative Transcriptome Sequencing. The transcriptome data in this study were more accurate. In procedure 5, after the first stage, we obtained 2546 DEGs; in the second stage, we obtained 2126 DEGs; in the third and fourth stages, we obtained 1402 and 2100 DEGs, respectively; and after transdifferentiation, relative to mature islets cells, we obtained 7387 DEGs. These results suggest that at the end of each transdifferentiation phase, the cells underwent corresponding changes and transdifferentiated toward IPCs, but a large gap with mature islet cells remained. Through GO functional enrichment analysis, we obtained 126 DEGs, and through KEGG pathway enrichment analysis, we obtained 266 DEGs and 18 pathways, all of which are related to islet development and insulin secretion. These data can be further mined and validated. Subsequently, through further bioinformatics analysis such as with protein interaction networks, we identified 18 genes as novel functional genes, which are of great significance for subsequent research.

### Novel Functional Genes Can Improve the Transdifferentiation Efficiency

In this study, we obtained 18 novel functional genes, and through verification, we found that 5 novel functional genes may be the key regulators of ADMSCs transdifferentiation into IPCs. Studies have shown that *Foxa1 Hnf1b*, *Dll1*, *Pbx1*, and *Rfx3* plays an important role in the regulatory network that controls the generation of pancreatic endocrine cell lines in model animals (Kim et al., 2002; Ait-Lounis et al., 2010; Gao et al., 2010; De Vas et al., 2015; Rubey et al., 2020). However, the roles of these genes in the transdifferentiation of ADMSCs into IPCs *in vitro* and whether these genes are key regulatory factors remain unknown. Therefore, the functioning of these five novel genes was verified by gene overexpression and silencing.

In overexpression experiments, the results showed that these five genes played an important role in the transdifferentiation of canine ADMSCs into IPCs. They can further improve insulin secretion and glucose SI, significantly improve the transdifferentiation efficiency and IPC maturity, with the most significant effects with *Foxa1*, *Pbx1*, and *Rfx3*. At the same time, the expression of islet development cascade regulation genes was significantly increased, but the specific direct or indirect effects need more research to verify. The solution of these problems can clarify the mechanism of these five genes improving transdifferentiation efficiency. After silencing these five genes, respectively, the transdifferentiation efficiency decreased to varying degrees, with silencing of *Foxa1*, *Pbx1*, and *Rfx3* genes showing significant decrease. This finding may be observed because *Dll1* and *Hnf1b*, when silenced, stimulate cascade regulatory gene expression in other ways; however, after *Foxa1*, *Pbx1*, and *Rfx3* were silenced, no other compensation appeared. This finding also proves the importance of *Foxa1*, *Pbx1*, and *Rfx3* in the transdifferentiation of ADMSCs into IPCs.

The above results prove that the five novel functional genes screened by us are of great significance in transdifferentiating ADMSCs into IPCs *in vitro*; *Foxa1*, *Pbx1*, and *Rfx3* are especially essential in the transdifferentiation of ADMSCs into IPCs and can be used as specifically key regulatory factors. In this study, after the overexpression/silencing of *Foxa1*, *Pbx1*, and *Rfx3*, we continued to conduct *in vitro* induction to explore the role of these three genes in the transdifferentiation of ADMSCs to IPCs. We will continue to explore the effects of *Foxa1*, *Pbx1*, and *Rfx3* on the function of mature islet β cells in future studies.

## Conclusion

In this study, canine ADMSCs were transdifferentiated into IPCs using five types of procedures, and the optimal procedure was determined. Many genes and signaling pathways were identified may play an important role in transdifferentiation of ADMSCs into IPCs in Absolute Quantitative Transcriptome Sequencing Analysis. *Hnf1B*, *Dll1*, *Pbx1*, *Rfx3*, and *Foxa1* were found to play important roles in ADMSCs transdifferentiating into IPCs. *Foxa1*, *Pbx1*, *Rfx3* exerted the most significant effects and can be used as specific key regulatory factors in the transdifferentiating of ADMSCs into IPCs. This study establishes a foundation for the further acquisition of IPCs with high maturity.

## Data Availability Statement

The datasets presented in this study can be found in online repositories. The names of the repository/repositories and accession number(s) can be found in the article/Supplementary Material 18.

## Ethics Statement

The animal study was reviewed and approved by the Animal Ethical and Welfare Committee of Northwest Agriculture and Forest University (Approval No: 2020002).

## Author Contributions

PD: methodology, data curation, writing—original draft, writing-review, and editing. JL: methodology, data curation, and writing—original draft. YC: data curation and writing—original draft. LZ: data curation and writing—original draft. XZ: data curation. JW: data curation. GQ: data curation. YZ: funding acquisition, project administration, supervision, writing—review, and editing. All authors contributed to the article and approved the submitted version.

## Conflict of Interest

The authors declare that the research was conducted in the absence of any commercial or financial relationships that could be construed as a potential conflict of interest.

## Footnotes

1 https://www.cyagen.com/cn/zh-cn/product/msc-adipogenic-differentiation-media-CAXMD-90031.html?from=34

2 https://www.cyagen.com/cn/zh-cn/product/msc-osteogenic-differentiation-media-CAXMD-90021.html?from=33

3 https://www.cyagen.com/cn/zh-cn/product/msc-chondrogenic-differentiation-media-CAXMD-90041.html?from=35

4 https://www.ncbi.nlm.nih.gov/genome/?term=Canis%20lupus%20familiaris

5 https://cran.rstudio.com/web/packages/pheatmap/index.html

6 http://geneontology.org

7 http://www.kegg.jp/kegg

## Supplementary Material

Supplementary Material 1 Five types of procedures.

Supplementary Material 2 Canine ADMSC isolation, culture, and identification.

Supplementary Material 3 All tables.

Supplementary Material 4 Quality control of sequencing data and the alignment with the reference genome.

Supplementary Material 5 The regional distribution aligned with the reference genome.

Supplementary Material 6 The result of genes expression level analysis.

Supplementary Material 7 DEGs.

Supplementary Material 8 GO Functional Enrichment Analysis of DEGs.

Supplementary Material 9 The 20 most significant GO terms for each group.

Supplementary Material 10 DEGs involved in pancreatic development and insulin secretion.

Supplementary Material 11 The 20 most significant KEGG pathways for each group.

Supplementary Material 12 Enrichment Analysis of KEGG Pathways of DEGs.

Supplementary Material 13 Functional analysis.

Supplementary Material 14 Protein interaction network analysis.

Supplementary Material 15 Five novel functional genes (Foxa1, Hnf1b, Dll1, Pbx1, Rfx3) were screened by validation.

Supplementary Material 16 RT-qPCR Verification of Absolute Quantitative Transcriptome Sequencing.

Supplementary Material 17 The expression of the target genes and proteins was verified by RT-qPCR and Western-blot.

Supplementary Material 18 The names of the repository/repositories and accession number(s).
