# Supplementary material for: Novel Functional Genes Involved in Transdifferentiation of Canine ADMSCs Into Insulin-Producing Cells, as Determined by Absolute Quantitative Transcriptome Sequencing Analysis
Source: Front Cell Dev Biol. 2021 Jun 28;9:685494. doi: 10.3389/fcell.2021.685494 (PMC8273515; doi:10.3389/fcell.2021.685494)
Supplement: Supplementary Material 1 — Five types of procedures. [file Data_Sheet_1.zip › Supplement 1.docx]

**Five Types of Procedures**

**Procedure 1:**

Day 1 to day 6: High Glucose Dulbecco's Modified Eagle Medium (H-DMEM) with 20 ng/mL Activin A, 300 ng/mL ATRA, 2% B27 and 0.5% BSA, the culture medium was changed every 2 days.

Day 7 to day 12: H-DMEM with 50 ng/mL EGF, 10 ng/mL bFGF, 2% B27 and 0.5% BSA, the culture medium was changed every 2 days.

Day 13 to day 18: Low Glucose Dulbecco's Modified Eagle Medium (L-DMEM) with 10 ng/mL BTC, 20 ng/mL GLP-1, 10 ng/mL Nicotinamide, 2% B27 and 0.5% BSA, the culture medium was changed every 2 days.

The cells were tested after 18 days.

**Procedure 2 :**

Day 1 to day 6: H-DMEM with 50 ng/mL EGF, 10 ng/mL bFGF, 2% B27 and 0.5% BSA, the culture medium was changed every 2 days.

Day 7 to day 12: H-DMEM with 200 ng/mL 5-AZA, 30 ng/mL TSA, 20 ng/mL Activin A, 2% B27 and 0.5% BSA, the culture medium was changed every 2 days.

Day 13 to day 18: L-DMEM with 300 ng/mL ATRA, 10 ng/mL Nicotinamide, 10 ng/mL Exendin-4, 2% B27 and 0.5% BSA, the culture medium was changed every 2 days.

The cells were tested after 18 days.

**Procedure 3 :**

Day 1 to day 6: H-DMEM with 10 ng/mL EGF, 10 ng/mL bFGF, 2% B27 and 0.5% BSA, the culture medium was changed every 2 days.

Day 7 to day 12: H-DMEM with 10 ng/mL EGF, 20 ng/mL Activin A, 10 ng/mL Nicotinamide, 2% B27 and 0.5% BSA, the culture medium was changed every 2 days.

Day 13 to day 18: L-DMEM with 10 ng/mL EGF, 10 ng/mL Exendin-4, 10 ng/mL BTC, 2% B27 and 0.5% BSA, the culture medium was changed every 2 days.

The cells were tested after 18 days.

**Procedure 4 :**

Day 1 to day 2: L-DMEM with 0.5% FBS, 20 ng/mL Activin A, 1.4 μg/mL Chir99021.

Day 3 to day 5: L-DMEM with 2% FBS, 10 ng/mL KGF, 100 ng/mL Sant1, 600 ng/mL ATRA, 85 ng/mL LDN193189, 260 ng/mL PdBU.

Day 6 to day 11: L-DMEM with 1% BSA, 10 ng/mL KGF, 100 ng/mL Sant1, 30 ng/mL ATRA, the culture medium was changed every 2 days.

Day 12 to day 15: H-DMEM with 1% BSA, 100 ng/mL Sant1, 30 ng/mL ATRA, 50 ng/mL XXI, 3 μg/mL Alk5i II, 650 ng/mL T3, 20 ng/mL BTC and 10 μg/mL Heparin, the culture medium was changed every 2 days.

Day 16 to day 19: H-DMEM with 1% BSA, 7.5 ng/mL ATRA, 50 ng/mL XXI, 3 μg/mL Alk5i II, 650 ng/mL T3, 20 ng/mL BTC and 10 μg/ml Heparin, the culture medium was changed every 2 days.

Day 20 to day 25: H-DMEM with 1% BSA, 3 μg/mL Alk5i II and 650 ng/mL T3, the culture medium was changed every 2 days.

The cells were tested after 25 days.

**Procedure 5 :**

Day 1 to day 2: L-DMEM with 0.5% BSA, 20 ng/mL GDF8 and 1.4 μg/mL Chir99021.

Day 3 to day 5: L-DMEM with 2% BSA, 45 μg/mL Ascorbic Acid, 10 ng/mL FGF7, 100 ng/mL Sant1, 300 ng/mL ATRA, 45 ng/mL LDN193189, 1:200 ITS-X and 100 ng/mL PdBU.

Day 6 to day 8: L-DMEM with 1% BSA, 45 μg/mL Ascorbic Acid, 2 ng/mL FGF7, 100 ng/ml Sant1, 30 ng/mL ATRA, 85 ng/mL LDN193189, 1:200 ITS-X and 50 ng/mL PdBU.

Day 9 to day 11: H-DMEM with 1% BSA, 100 ng/mL Sant1, 15 ng/mL ATRA, 45 ng/mL LDN193189, 1:200 ITS-X, 650 ng/mL T3, 3 μg/mL Alk5i II, 1.6 μg/mL Zinc Sulfate and 10 μg/mL Heparin.

Day 12 to day 15: H-DMEM with 1% BSA, 45 ng/mL LDN193189, 1:200 ITS-X, 650 ng/mL T3, 3 μg/mL Alk5i II, 1.6 μg/mL Zinc Sulfate, 50 ng/mL XXI and 10 μg/mL Heparin, the culture medium was changed every 2 days.

Day16 to day 19: H-DMEM with 1% BSA, 45 ng/mL LDN193189, 1:200 ITS-X, 650 ng/mL T3, 3 μg/mL Alk5i II, 1.6 μg/mL Zinc Sulfate and 10 μg/mL Heparin, the culture medium was changed every 2 days.

Day 20 to day 25: H-DMEM with 1% BSA, 1:200 ITS-X, 650 ng/mL T3, 3 μg/mL Alk5i II, 1.6 μg/mL Zinc Sulfate, 160 μg/mL N-acetyl Cysteine, 2.5 μg/mL Trolox, 1 μg/mL R428 and 10 μg/mL Heparin, the culture medium was changed every 2 days.

The cells were tested after 25 days.
