# Supplementary material for: Novel Functional Genes Involved in Transdifferentiation of Canine ADMSCs Into Insulin-Producing Cells, as Determined by Absolute Quantitative Transcriptome Sequencing Analysis
Source: Front Cell Dev Biol. 2021 Jun 28;9:685494. doi: 10.3389/fcell.2021.685494 (PMC8273515; doi:10.3389/fcell.2021.685494)
Supplement: Supplementary Material 1 — Five types of procedures. [file Data_Sheet_1.zip › Supplement 10.docx]

**DEGs involved in pancreatic development and insulin secretion**

| **All DEGs** | **DEGs of IPC1VSADSC** | **DEGs of IPC2VSIPC1** | **DEGs of IPC3VSIPC2** | **DEGs of IPC4VSIPC3** | **DEGs of Beta_cellVSIPC4** | **GO_Term** |
| --- | --- | --- | --- | --- | --- | --- |
| *Eif2ak3; Foxa2; Gipr; Hnf1a; Hnf1b; Il6r; Il6; Mnx1; Neurod1; Nkx6-1; Onecut1; Onecut2; Sox4; Sox9* | *Sox4* | *Il6r*  *Il6*  *Sox4*  *Sox9* | *Il6;*  *Sox4* | *Il6* | *Foxa2*  *Gipr*  *Hnf1a*  *Hnf1b*  *Il6r*  *Mnx1*  *Neurod1*  *Nkx6-1*  *Onecut1*  *Onecut2*  *Sox4*  *Sox9* | endocrine pancreas development |
| *Cela1; Loc102154267; Nr5a2* |  |  | *Cela1* | *Cela1* | *Cela1*  *Loc102154267*  *Nr5a2* | pancreas morphogenesis |
| *Acvr2b; Aldh1a2; Ctnnb1; Foxf1; Gdf11; Hhex; Ildr2; Isl1; Meis2; Ptf1a; Selenot; Smad2* | *Foxf1*  *Ildr2* | *Hhex*  *Ildr2* |  | *Ildr2* | *Aldh1a2*  *Ctnnb1*  *Foxf1*  *Gdf11*  *Hhex*  *Isl1*  *Ptf1a*  *Smad2* | pancreas development |
| *Prox1* |  |  | *Prox1* |  | *Prox1* | branching involved in pancreas morphogenesis |
| *Adcy5; Birc5; Brsk2; Cdk16; Uba1; Efna5; Epha5; Hmgn3; Lrp5; Nr1d1; Nrxn1; Prkce; Sidt2; Stxbp4* | *Adcy5*  *Birc5*  *Efna5* | *Lrp5* | *Adcy5* | *Birc5* | *Brsk2*  *Cdk16*  *Uba1*  *Epha5*  *Hmgn3*  *Lrp5*  *Nr1d1*  *Nrxn1*  *Sidt2* | regulation of insulin secretion involved in cellular response to glucose stimulus |
| *Acvr2b; Cplx1; Cyb5r4; Fam3b; Ffar1; Ffar3; Fkbp1b; Gpr119; Hnf1a; Hnf1b; Il1rn; Ildr2; Lep; Loc111090913; Neurod1; Pclo; Pdx1; Ptprn; Rims2; Slc30a8; Smad2; Snx19; Stxbp3; Vgf* |  | *Ffar3*  *Ffar1*  *Ildr2*  *Pclo*  *Ptprn* | *Fkbp1b*  *Pclo*  *Ptprn* | *Ffar3*  *Ffar1*  *Fkbp1b*  *Ildr2*  *Ptprn* | *Fam3b*  *Ffar1*  *Ffar3*  *Gpr119*  *Hnf1a*  *Hnf1b*  *Il1rn*  *Lep*  *Loc111090913*  *Neurod1*  *Pclo*  *Pdx1*  *Rims2*  *Slc30a8*  *Smad2;Vgf* | insulin secretion |
| *Ano1; Arrb1; Bad; C2cd2l; Cftr; Gcg; Gpr68; Hif1a; Mpc2; Pdx1; Ppp3cb; Rfx6; Sri; Stx4; Tmem27* | *Ano1*  *Arrb1*  *Gpr68* | *Arrb1*  *C2cd2l*  *Gpr68* | *Arrb1* | *Arrb1*  *Gpr68* | *Ano1*  *Arrb1*  *Cftr*  *Gcg*  *Gpr68*  *Hif1a*  *Mpc2*  *Pdx1*  *Rfx6*  *Stx4*  *Tmem27* | positive regulation of insulin secretion involved in cellular response to glucose stimulus |
| *Ptprn2; Rab11b; Rab11fip2; Rab11fip5; Raf1; Selenot* |  |  |  |  | *Ptprn2*  *Rab11fip2*  *Rab11fip5*  *Raf1* | insulin secretion involved in cellular response to glucose stimulus |
| *Capn10; Doc2b; Ffar1; Ffar3; Gck; Gper1; Irs2; Mcu; Nkx6-1; Nlgn2; Nnat; Pfkm; Prkce; Rbp4; Sox4; Tcf7l2* | *Doc2b*  *Gper1*  *Rbp4*  *Sox4* |  | *Nnat*  *Rbp4*  *Sox4* | *Doc2b*  *Ffar3*  *Ffar1*  *Nnat* | *Doc2b*  *Ffar1*  *Ffar3*  *Gck*  *Gper1*  *Nkx6-1*  *Nlgn2*  *Pfkm*  *Rbp4*  *Sox4*  *Tcf7l2* | positive regulation of insulin secretion |
| *Arl2; Arntl; Cacna2d2; Ccl5; Clock; Ensa; G6pc2; Gck; Hnf1a; Hnf4a; Ica1; Lep; Loc100686848; Nkx6-1; Nos2; Rfx3; Rfx6; Slc16a1; Syt7; Syt9; Tfap2b* | *Arntl*  *Ccl5*  *Ica1*  *Nos2*  *Rfx3* |  | *Cacna2d2*  *Syt9* | *Adra2a*  *Fkbp1b*  *Loc100682773*  *Map4k4* | *Cacna2d2*  *Ccl5*  *Clock*  *Ensa*  *G6pc2*  *Gck*  *Hnf1a*  *Hnf4a*  *Ica1*  *Lep*  *Loc100686848*  *Nkx6-1*  *Rfx3*  *Rfx6*  *Slc16a1*  *Syt7* | regulation of insulin secretion |
