# Supplementary material for: Novel Functional Genes Involved in Transdifferentiation of Canine ADMSCs Into Insulin-Producing Cells, as Determined by Absolute Quantitative Transcriptome Sequencing Analysis
Source: Front Cell Dev Biol. 2021 Jun 28;9:685494. doi: 10.3389/fcell.2021.685494 (PMC8273515; doi:10.3389/fcell.2021.685494)
Supplement: Supplementary Material 1 — Five types of procedures. [file Data_Sheet_1.zip › Supplement 13.docx]

A total of 51 genes that we screened

1 Wnt5a protein Wnt-5a isoform X2 [Lagenorhynchus obliquidens]

2 Sox4 transcription factor SOX-4 [Canis lupus familiaris]

3 Sox8 transcription factor SOX-8 [Canis lupus familiaris]

4 Sox17 LOW QUALITY PROTEIN: transcription factor SOX-17 [Canis lupus familiaris]

5 Gdf11 growth/differentiation factor 11 isoform X1 [Canis lupus familiaris]

6 Cela1 chymotrypsin-like elastase family member 1 [Canis lupus dingo]

7 Foxo1 LOW QUALITY PROTEIN: forkhead box protein O1 [Canis lupus familiaris]

8 Foxa1 hepatocyte nuclear factor 3-alpha [Canis lupus familiaris]

9 Foxf1 LOW QUALITY PROTEIN: forkhead box protein F1 [Canis lupus familiaris]

10 Onecut1 hepatocyte nuclear factor 6 [Canis lupus familiaris]

11 Onecut2 one cut domain family member 2 [Canis lupus familiaris]

12 Bmp4 bone morphogenetic protein 4, isoform CRA_b, partial [Mus musculus]

13 Bmp5 bone morphogenetic protein 5 isoform X2 [Canis lupus familiaris]

14 Hnf1a hepatocyte nuclear factor 1-alpha isoform X1 [Canis lupus familiaris]

15 Hnf1b hepatocyte nuclear factor 1-beta isoform X1 [Canis lupus familiaris]

16 Hnf4a hepatocyte nuclear factor 4-alpha isoform X5 [Canis lupus familiaris]

17 Rfx8 DNA-binding protein RFX8 [Canis lupus familiaris]

18 Rfx6 DNA-binding protein RFX6 [Canis lupus familiaris]

19 Rfx3 transcription factor RFX3 isoform X9 [Canis lupus familiaris]

20 Hadh hydroxyacyl-coenzyme A dehydrogenase, mitochondrial [Canis lupus familiaris]

21 Ide insulin-degrading enzyme [Canis lupus familiaris]

22 Tcf7l2 transcription factor 7-like 2 isoform X10 [Canis lupus familiaris]

23 Nr5a2 nuclear receptor subfamily 5 group A member 2 isoform X1 [Vulpes vulpes]

24 Gck glucokinase [Canis lupus familiaris]

25 Pbx1 pre-B-cell leukemia transcription factor 1 isoform X4 [Canis lupus familiaris]

26 Dll1 delta-like protein 1, partial [Canis lupus familiaris]

27 Ptf1a LOW QUALITY PROTEIN: pancreas transcription factor 1 subunit alpha [Canis lupus familiaris]

28 Insrr insulin receptor-related protein isoform X2 [Canis lupus familiaris]

29 Insig1 PREDICTED: insulin-induced gene 1 protein [Panthera tigris altaica]

30 Insig2 insulin-induced gene 2 protein [Canis lupus familiaris]

31 Il6 interleukin-6 [Canis lupus dingo]

32 Il6r interleukin-6 receptor subunit alpha isoform X3 [Canis lupus familiaris]

33 Ildr2 immunoglobulin-like domain-containing receptor 2 isoform X2 [Canis lupus familiaris]

34 Meis2 PREDICTED: homeobox protein Meis2 isoform X2 [Mustela putorius furo]

35 Wls protein wntless homolog [Canis lupus familiaris]

36 Smad2 PREDICTED: mothers against decapentaplegic homolog 2 isoform X1 [Mustela putorius furo]

37 Hhex hematopoietically-expressed homeobox protein HHEX [Canis lupus familiaris]

38 Ppdpfl pancreatic progenitor cell differentiation and proliferation factor-like protein isoform X2 [Canis lupus familiaris]

39 Ctnnb1 Catenin beta-1 [Heterocephalus glaber]

40 Gata5 transcription factor GATA-5 [Canis lupus familiaris]

41 Gid8 glucose-induced degradation protein 8 homolog [Canis lupus familiaris]

42 Igf2 insulin-like growth factor 2 splice variant 2 [Canis lupus familiaris]

43 Gipr gastric inhibitory polypeptide receptor isoform X1 [Canis lupus familiaris]

44 Selenot selenoprotein T precursor [Canis lupus familiaris]

45 Eif2ak3 eukaryotic translation initiation factor 2-alpha kinase 3 isoform X1 [Canis lupus familiaris]

46 Acvr2b activin receptor type-2B isoform X2 [Canis lupus familiaris]

47 Vegfd vascular endothelial growth factor D [Canis lupus familiaris]

48 Vegfc vascular endothelial growth factor C [Canis lupus familiaris]

49 Vegfb vascular endothelial growth factor B isoform X1 [Vulpes vulpes]

50 Vegfa vascular endothelial growth factor A [Neomonachus schauinslandi]

51 Insm1 insulinoma-associated protein 1 [Canis lupus familiaris]
