# Supplementary material for: Novel Functional Genes Involved in Transdifferentiation of Canine ADMSCs Into Insulin-Producing Cells, as Determined by Absolute Quantitative Transcriptome Sequencing Analysis
Source: Front Cell Dev Biol. 2021 Jun 28;9:685494. doi: 10.3389/fcell.2021.685494 (PMC8273515; doi:10.3389/fcell.2021.685494)
Supplement: Supplementary Material 1 — Five types of procedures. [file Data_Sheet_1.zip › Supplement 18.docx]

Sequence data were deposited in the NCBI Short Read Archive database (BioProject accession number: PRJNA647127; BioSample accession number: SAMN15576633 and SAMN15576634; SRA accession numbers: SRR12277423, SRR12277425, SRR12277418, SRR12277422, SRR12277421, SRR12277420, SRR12277424 and SRR12277419.
