# Supplementary material for: Novel Functional Genes Involved in Transdifferentiation of Canine ADMSCs Into Insulin-Producing Cells, as Determined by Absolute Quantitative Transcriptome Sequencing Analysis
Source: Front Cell Dev Biol. 2021 Jun 28;9:685494. doi: 10.3389/fcell.2021.685494 (PMC8273515; doi:10.3389/fcell.2021.685494)
Supplement: Supplementary Material 1 — Five types of procedures. [file Data_Sheet_1.zip › Supplement 3.docx]

**Table 1 The primers of RT-qPCR ( islet β-cell related genes)**

| **Name of the primers** | **Sequences of primers** |
| --- | --- |
| PDX1-F | GTGGATGAAGTCTACCAAGG |
| PDX1-R | TTGAACAGGAACTCCTTCTC |
| MAFA-F | AGCAAGGAGGAGGTCATC |
| MAFA-R | CTTCTCGCTCTCCAGGAT |
| NKX6.1-F | GGAGAGTCAGGTCAAGGT |
| NKX6.1-R | GTTGTAGTCGTCGTCCTC |
| INS-F | GCTTCTTCTACACGCCTAA |
| INS-R | CTAGTTGCAGTAATTCTCCAG |
| PCSK1-F | AGTGGAGAAGATGGTGGAT |
| PCSK1-R | TTGTAGGAGTCTAAGCATAGC |
| NKX2.2-F | CTTCTCCAAGGCACAGAC |
| NKX2.2-R | TCTTGTAGCGGTGGTTCT |
| PAX4-F | GGAGACATCACCAGACAG |
| PAX4-R | AATGGAGGCAATGGAAGG |

**Table 2 The primers of RT-qPCR (RT-qPCR Verification of Absolute Quantitative Transcriptome Sequencing)**

| **Name of the primers** | **Sequences of primers** |
| --- | --- |
| YBMP4-F | GCTGGAATGACTGGATTGT |
| YBMP4-R | CGTGGTTGGTTGAGTTGA |
| YDLL1-F | GTGTGATGAGCATTACTACG |
| YDLL1-R | GCAAGCAGATTGGTTCAG |
| YFOXO1-F | GACATAGCCAGCCAACAT |
| YFOXO1-R | CCATCCTTCCATAGCCATT |
| YHNF1A-F | AGAGGTGCGTGTCTACAA |
| YHNF1A-R | AGTTGCTTGCTGTCCATAG |
| YPBX1-F | GCTGTCACTGCTACCAAT |
| YPBX1-R | TCCTGCCAACCTCCATTA |
| YTCF7L2-F | CACCACATCACACTCTACA |
| YTCF7L2-R | TTCGCTTGCTCTTCTCTG |
| YWNT5A-F | GCAATGTCTTCCAAGTTCTT |
| YWNT5A-R | TGATACAAGTGGCACAGTT |
| YGATA-4-F | CAGCAACTCCAGCAATGT |
| YGATA-4-R | ATCGCACTGACTGAGAATG |
| YRFX3-F | GCTATTCTCCTTCTACTCCAA |
| YRFX3-R | GGTCACAGTTACACATCCA |
| YISL1-F | CAAGAAGCGGAGCATCAT |
| YISL1-R | AGGCGAAGTCACTCAGTA |
| YPCSK2-F | CCGTGTATCTCAGTGGAAG |
| YPCSK2-R | GCTGCTGCTTGTGATGTA |
| YSOX9-F | CACATCAAGACGGAGCAG |
| YSOX9-R | AGGTGAAGGTGGAGTAGAG |

**Table 3 The primers** **of genes (five genes were amplified by PCR)**

| **Name of the primers** | **Sequences of primers** |
| --- | --- |
| FOXA1-F | gtcagatccgctagagatctGCCACCATGCTGGGCACCGTGAAGATGG |
| FOXA1-R | gatatcttatctagaagcttCTACAGCTGCTTGGTGCCCTTT |
| DLL1-F | gtcagatccgctagagatctGCCACCATGGGCGACAGAAATTGCTGTAGAG |
| DLL1-R | gatatcttatctagaagcttTCAAACTTCGGTGGCGATCACG |
| HNF1B-F | gtcagatccgctagagatctGCCACCATGGTGTCCAAGCTGACCAGCC |
| HNF1B-R | gatatcttatctagaagcttTCACCAAGCCTGCAGTGGGCAC |
| PBX1-F | gtcagatccgctagagatctGCCACCATGGACGAGCAGCCCAGACTGA |
| PBX1-R | gatatcttatctagaagcttTCAGTTAGAGGTATCAGAGTGC |
| RFX3-F | gtcagatccgctagagatctGCCACCATGCAGACCAGCGAAACCGGCT |
| RFX3-R | gatatcttatctagaagcttTCAGCCGAAGGGGATTGTCTCT |

Underline base is the homologous arm

**Table 4 The primers of RT-qPCR (the expression of the target genes verified by RT-qPCR)**

| **Name of the primers** | **Sequences of primers** |
| --- | --- |
| QFOXA1-F | CGGCTCTATGAACTCTATGA |
| QFOXA1-R | CTTGAAGGTCTTGGCATCT |
| QDLL1-F | GGATTCAGTGGCAGACAG |
| QDLL1-R | TCGCACAGGTATCTTCTATC |
| QHNF1B-F | TGTCTGCTCTGCTGTCTA |
| QHNF1B-R | ATCTCCGCTCAGTCTACC |
| QPBX1-F | CGTGATGAACCTGCTGAG |
| QPBX1-R | TCTGGATGCTGCTGAACT |
| QRFX3-F | AACAACCATCACCGAGAG |
| QRFX3-R | CAGCCTTCCAGAGACTTG |

**Table 5 The SiRNA sequences**

| **Name** | **21nt passenger (5′→3′)** | **21nt guide (5′→3′)** |
| --- | --- | --- |
| FOXA1 FAM-SiRNA-1 | CCUACUACCGGCAGAAUCAGC | UGAUUCUGCCGGUAGUAGGGG |
| FOXA1 FAM-SiRNA-2 | CGAAUUUCUCUGGUUUUCACC | UGAAAACCAGAGAAAUUCGCC |
| FOXA1 FAM-SiRNA-3 | GCUACAAACGCUUAAGUCAGC | UGACUUAAGCGUUUGUAGCUG |
| HNF1B FAM-SiRNA-1 | GAAAGCAACGAGAGAUUCUCC | AGAAUCUCUCGUUGCUUUCGG |
| HNF1B FAM-SiRNA-2 | GCUACAUGCAGCAACACAACA | UUGUGUUGCUGCAUGUAGCCC |
| HNF1B FAM-SiRNA-3 | GCAGAACUCCCACAUGUAUAC | AUACAUGUGGGAGUUCUGCAG |
| DLL1 FAM-SiRNA-1 | CCUUUUCUCUGAUCAUUGAAG | UCAAUGAUCAGAGAAAAGGUG |
| DLL1 FAM-SiRNA-2 | CAGAAAACCCAGAAAGACUCA | AGUCUUUCUGGGUUUUCUGUU |
| DLL1 FAM-SiRNA-3 | GGUGUAUUCCACUUCGAAAGA | UUUCGAAGUGGAAUACACCGA |
| PBX1 FAM-SiRNA-1 | CCAGAAAACAUGCUUUAAACU | UUUAAAGCAUGUUUUCUGGCC |
| PBX1 FAM-SiRNA-2 | GACAUUUUACAGCAAAUUAUG | UAAUUUGCUGUAAAAUGUCUC |
| PBX1 FAM-SiRNA-3 | GGACAUUGGAGACAUUUUACA | UAAAAUGUCUCCAAUGUCCUG |
| RFX3 FAM-SiRNA-1 | GAACAACAACUUAUCCUUACA | UAAGGAUAAGUUGUUGUUCGG |
| RFX3 FAM-SiRNA-2 | GCUUCUUUUGGAAAACUAAUA | UUAGUUUUCCAAAAGAAGCAG |
| RFX3 FAM-SiRNA-3 | CUUCUUUUGGAAAACUAAUAA | AUUAGUUUUCCAAAAGAAGCA |
| Negative control FAM-SiRNA | UUCUCCGAACGUGUCACGUTT | ACGUGACACGUUCGGAGAATT |
