# Supplementary material for: Novel Functional Genes Involved in Transdifferentiation of Canine ADMSCs Into Insulin-Producing Cells, as Determined by Absolute Quantitative Transcriptome Sequencing Analysis
Source: Front Cell Dev Biol. 2021 Jun 28;9:685494. doi: 10.3389/fcell.2021.685494 (PMC8273515; doi:10.3389/fcell.2021.685494)
Supplement: Supplementary Material 1 — Five types of procedures. [file Data_Sheet_1.zip › Supplement 4.docx]

**Table 1 Overview of Quality control of sequencing data**

| **Sample** | **Raw_reads** | **Raw_bases(G)** | **Valid_reads** | **Dedup_reads** | **Valid_Q20(%)** | **Valid_Q30(%)** | **Valid_GC(%)** | **Valid2raw(%)** | **Dedup2Valid(%)** |
| --- | --- | --- | --- | --- | --- | --- | --- | --- | --- |
| IPC1_1 | 48010724 | 7.20G | 46982516 | 38122833 | 99.95% | 96.86% | 48.5% | 97.86% | 81.14% |
| IPC1_2 | 46500688 | 6.98G | 45485702 | 36766168 | 99.95% | 96.76% | 49% | 97.82% | 80.83% |
| IPC2_1 | 55717400 | 8.36G | 54606364 | 43140682 | 99.95% | 96.83% | 49.5% | 98.01% | 79.00% |
| IPC2_2 | 46094966 | 6.91G | 45229606 | 37207952 | 99.95% | 96.58% | 49.5% | 98.12% | 82.26% |
| IPC3_1 | 37512440 | 5.63G | 36444940 | 32319789 | 99.95% | 96.17% | 50% | 97.15% | 88.68% |
| IPC3_2 | 38144814 | 5.72G | 36802080 | 32794492 | 99.95% | 95.98% | 49.5% | 96.48% | 89.11% |
| IPC4_1 | 37183910 | 5.58G | 36064364 | 32148798 | 99.95% | 95.75% | 50.5% | 96.99% | 89.14% |
| IPC4_2 | 44254668 | 6.64G | 43127438 | 36558967 | 99.95% | 96.65% | 49.5% | 97.45% | 84.77% |
| Beta_cell1 | 40340256 | 6.05G | 39458314 | 34585827 | 99.96% | 95.98% | 51.5% | 97.81% | 87.65% |
| Beta_cell2 | 47246484 | 7.09G | 46273036 | 37719019 | 99.96% | 96.63% | 51% | 97.94% | 81.51% |
| ADSC1 | 46393960 | 6.96G | 45519392 | 36800712 | 99.95% | 96.24% | 49.5% | 98.11% | 80.85% |
| ADSC2 | 49160866 | 7.37G | 47969462 | 39576021 | 99.95% | 96.74% | 49.5% | 97.58% | 82.50% |

**Table 2 The alignment with the reference genome**

| **Sample** | **Valid reads** | **Mapped reads** | **Unique Mapped reads** | **Multi Mapped reads** | **PE Mapped reads** | **Reads map to sense strand** | **Reads map to antisense strand** | **Non-splice reads** | **Splice reads** |
| --- | --- | --- | --- | --- | --- | --- | --- | --- | --- |
| IPC1_1 | 46982516 | 45297416(96.41%) | 36383129(77.44%) | 8914287(18.97%) | 43636402(92.88%) | 18389982(39.14%) | 18414691(39.19%) | 19551886(41.62%) | 17252787(36.72%) |
| IPC1_2 | 45485702 | 43821847(96.34%) | 35010896(76.97%) | 8810951(19.37%) | 42100208(92.56%) | 17729812(38.98%) | 17751519(39.03%) | 18929114(41.62%) | 16552217(36.39%) |
| IPC2_1 | 54606364 | 52624397(96.37%) | 42752472(78.29%) | 9871925(18.08%) | 50716458(92.88%) | 20878886(38.24%) | 20880016(38.24%) | 22617269(41.42%) | 19141633(35.05%) |
| IPC2_2 | 45229606 | 43518437(96.22%) | 34836716(77.02%) | 8681721(19.19%) | 41802976(92.42%) | 18013632(39.83%) | 18020520(39.84%) | 19484741(43.08%) | 16549411(36.59%) |
| IPC3_1 | 36444940 | 34952002(95.90%) | 27481506(75.41%) | 7470496(20.50%) | 33396060(91.63%) | 15612438(42.84%) | 15612956(42.84%) | 16697659(45.82%) | 14527735(39.86%) |
| IPC3_2 | 36802080 | 35245810(95.77%) | 27659972(75.16%) | 7585838(20.61%) | 33692318(91.55%) | 15849741(43.07%) | 15854707(43.08%) | 17199134(46.73%) | 14505314(39.41%) |
| IPC4_1 | 36064364 | 34483942(95.62%) | 26984266(74.82%) | 7499676(20.80%) | 33023872(91.57%) | 15520530(43.04%) | 15533997(43.07%) | 15656449(43.41%) | 15398078(42.70%) |
| IPC4_2 | 43127438 | 41540651(96.32%) | 33138551(76.84%) | 8402100(19.48%) | 39751240(92.17%) | 17669765(40.97%) | 17682720(41.00%) | 18573339(43.07%) | 16779146(38.91%) |
| Beta_cell1 | 39458314 | 38130140(96.63%) | 26434852(66.99%) | 11695288(29.64%) | 36890768(93.49%) | 15167788(38.44%) | 15129751(38.34%) | 10829321(27.44%) | 19468218(49.34%) |
| Beta_cell2 | 46273036 | 44712010(96.63%) | 31958587(69.07%) | 12753423(27.56%) | 43311542(93.60%) | 16566360(35.80%) | 16530204(35.72%) | 12329371(26.64%) | 20767193(44.88%) |
| ADSC1 | 45519392 | 43414075(95.37%) | 34175715(75.08%) | 9238360(20.30%) | 41771590(91.77%) | 17749795(38.99%) | 17781894(39.06%) | 17886457(39.29%) | 17645232(38.76%) |
| ADSC2 | 47969462 | 46180375(96.27%) | 36470936(76.03%) | 9709439(20.24%) | 44360288(92.48%) | 19096218(39.81%) | 19130884(39.88%) | 19441162(40.53%) | 18785940(39.16%) |
