# Supplementary figures and images for: Novel Functional Genes Involved in Transdifferentiation of Canine ADMSCs Into Insulin-Producing Cells, as Determined by Absolute Quantitative Transcriptome Sequencing Analysis
Source: Front Cell Dev Biol. 2021 Jun 28;9:685494. doi: 10.3389/fcell.2021.685494 (PMC8273515; doi:10.3389/fcell.2021.685494)

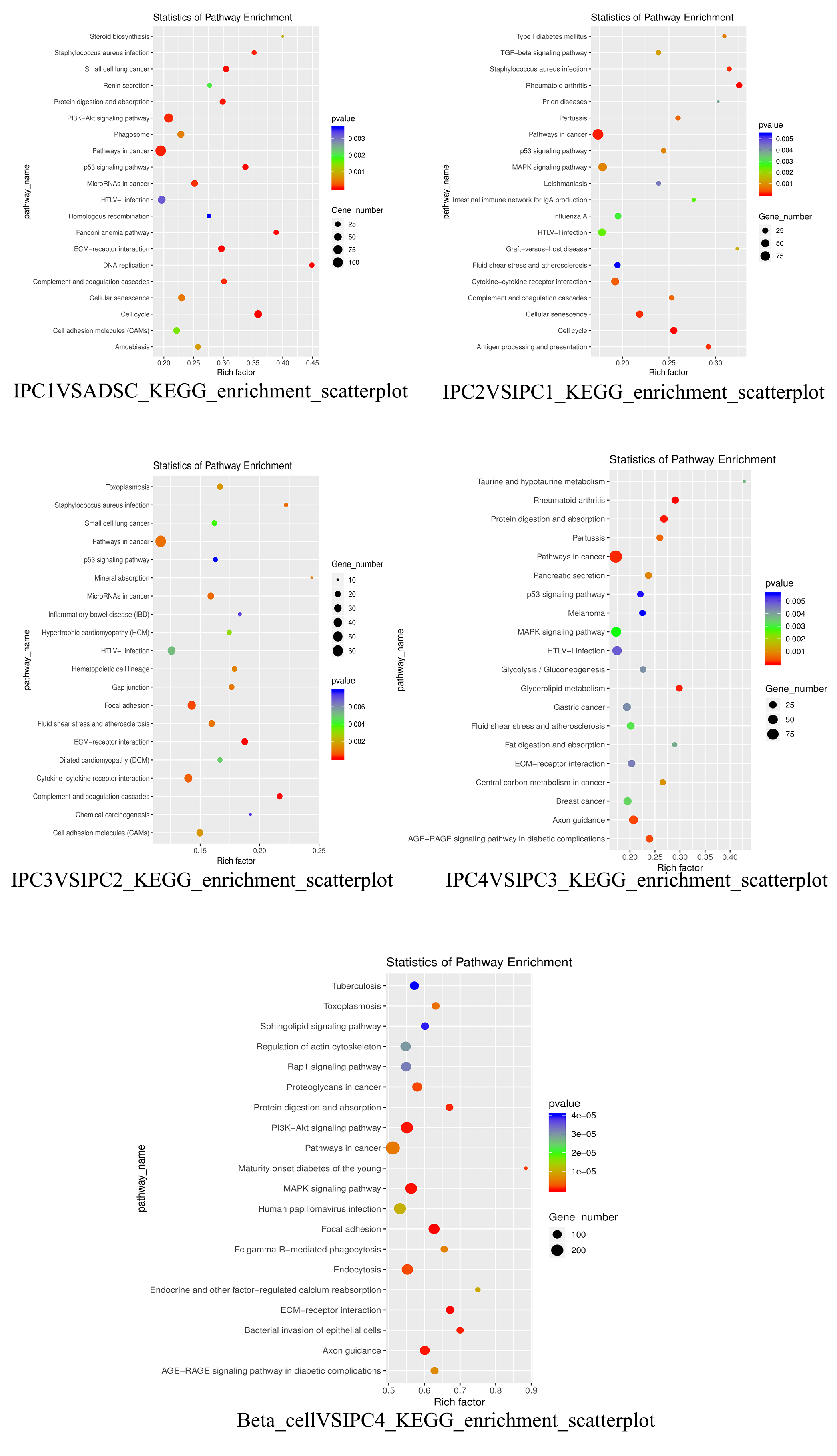

Supplement: Supplementary Material 1 — Five types of procedures. [file Data_Sheet_1.zip › Supplement 11.tif]

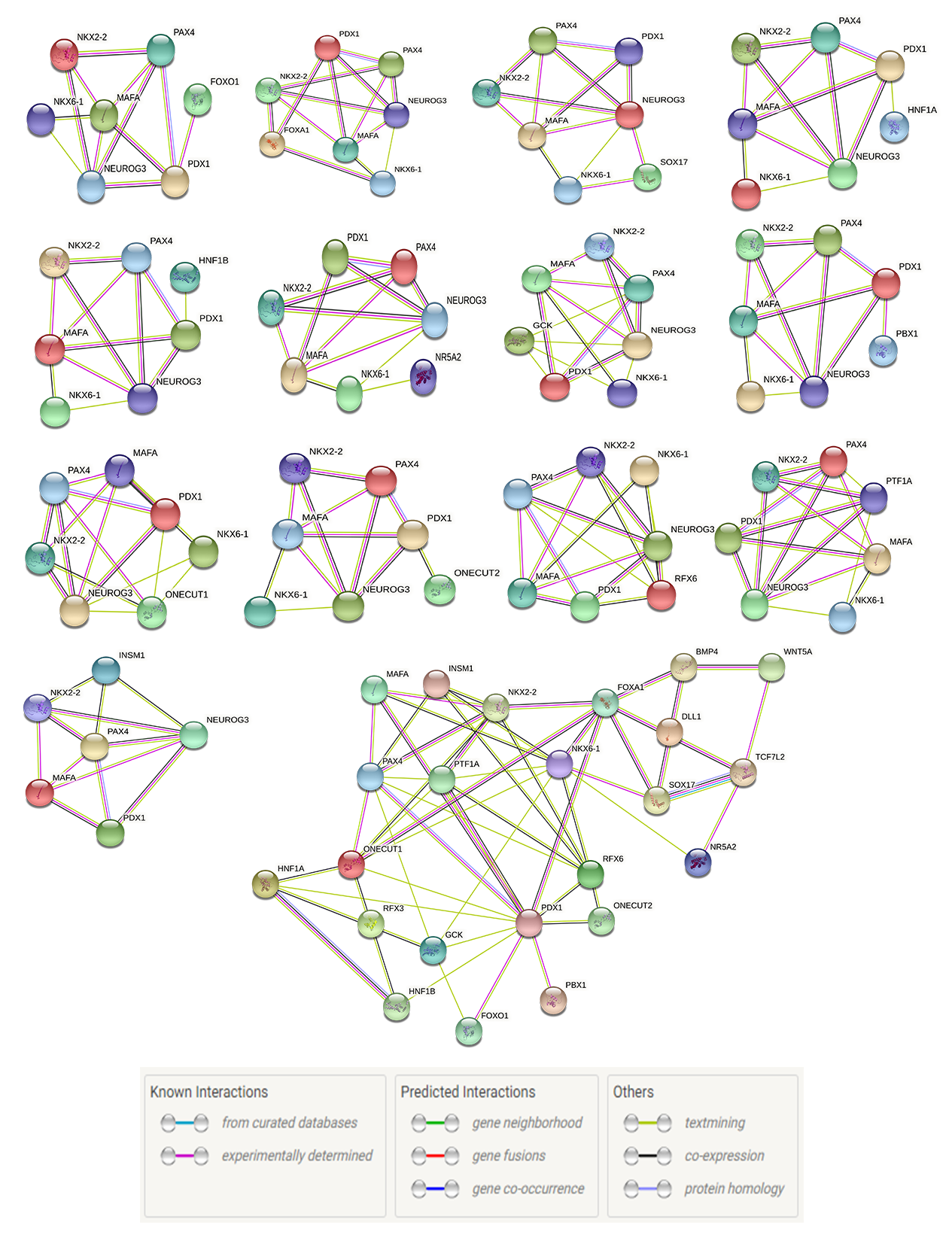

Supplement: Supplementary Material 1 — Five types of procedures. [file Data_Sheet_1.zip › Supplement 14.tif]

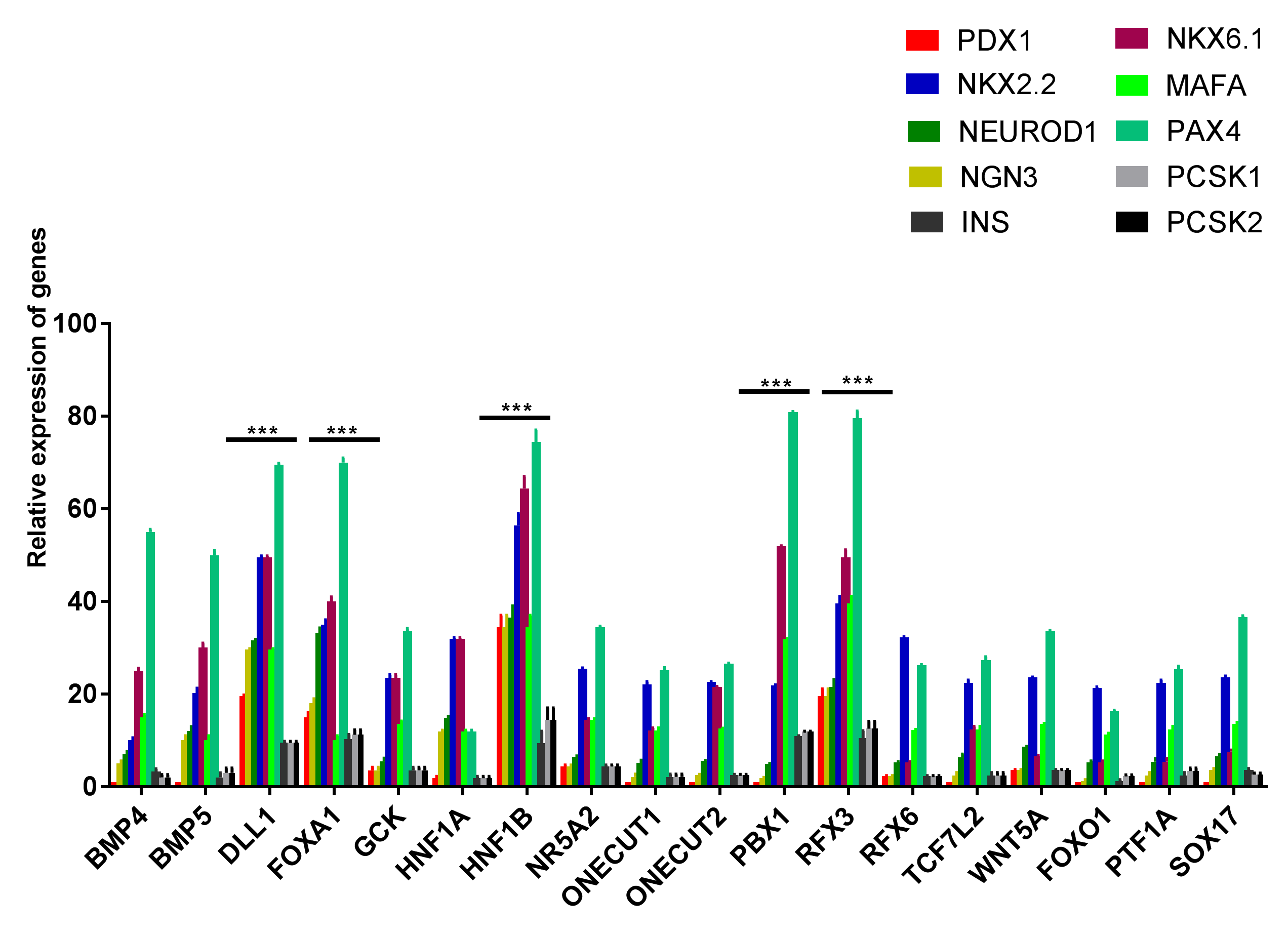

Supplement: Supplementary Material 1 — Five types of procedures. [file Data_Sheet_1.zip › Supplement 15.tif]

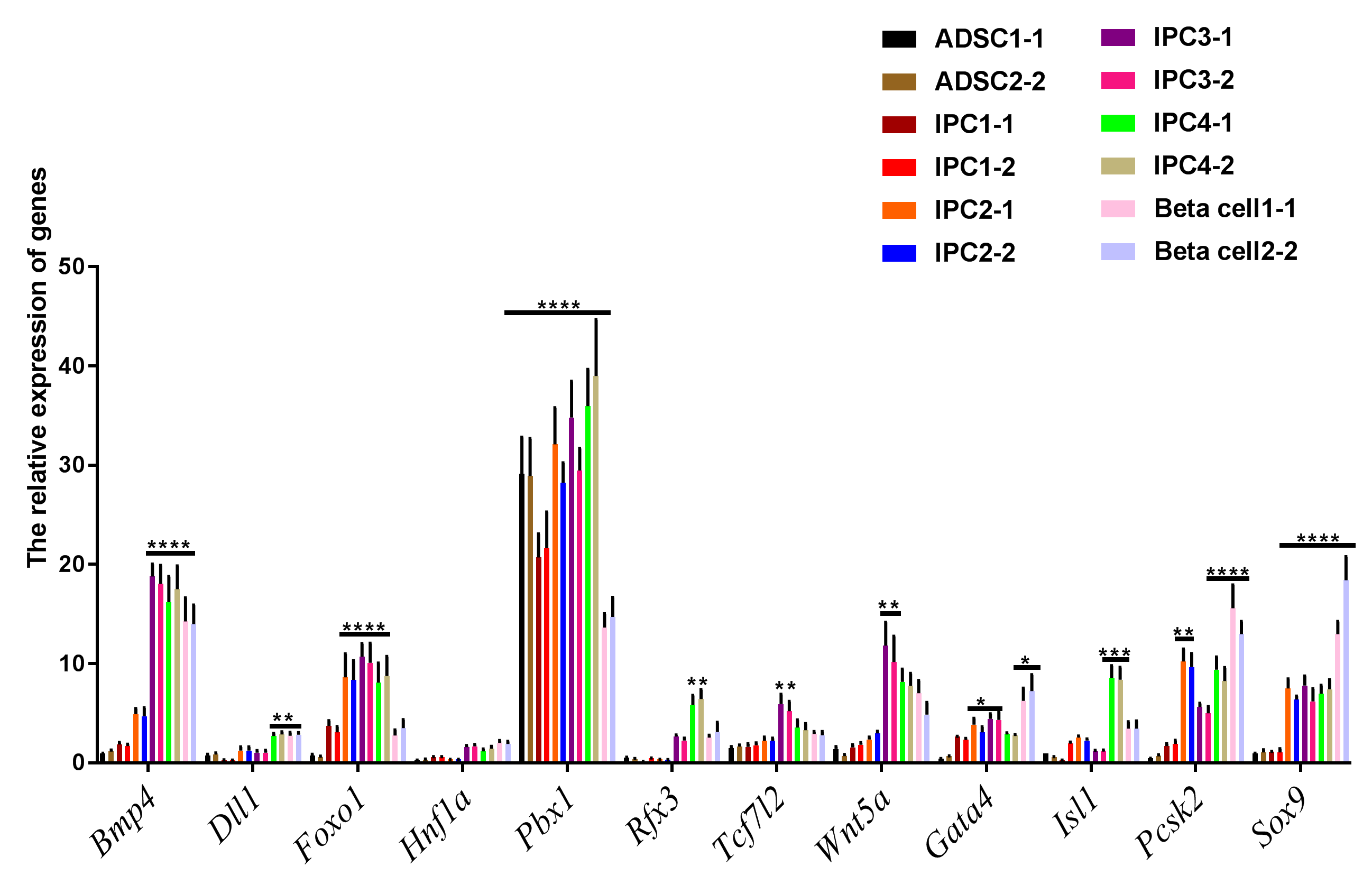

Supplement: Supplementary Material 1 — Five types of procedures. [file Data_Sheet_1.zip › Supplement 16.tif]

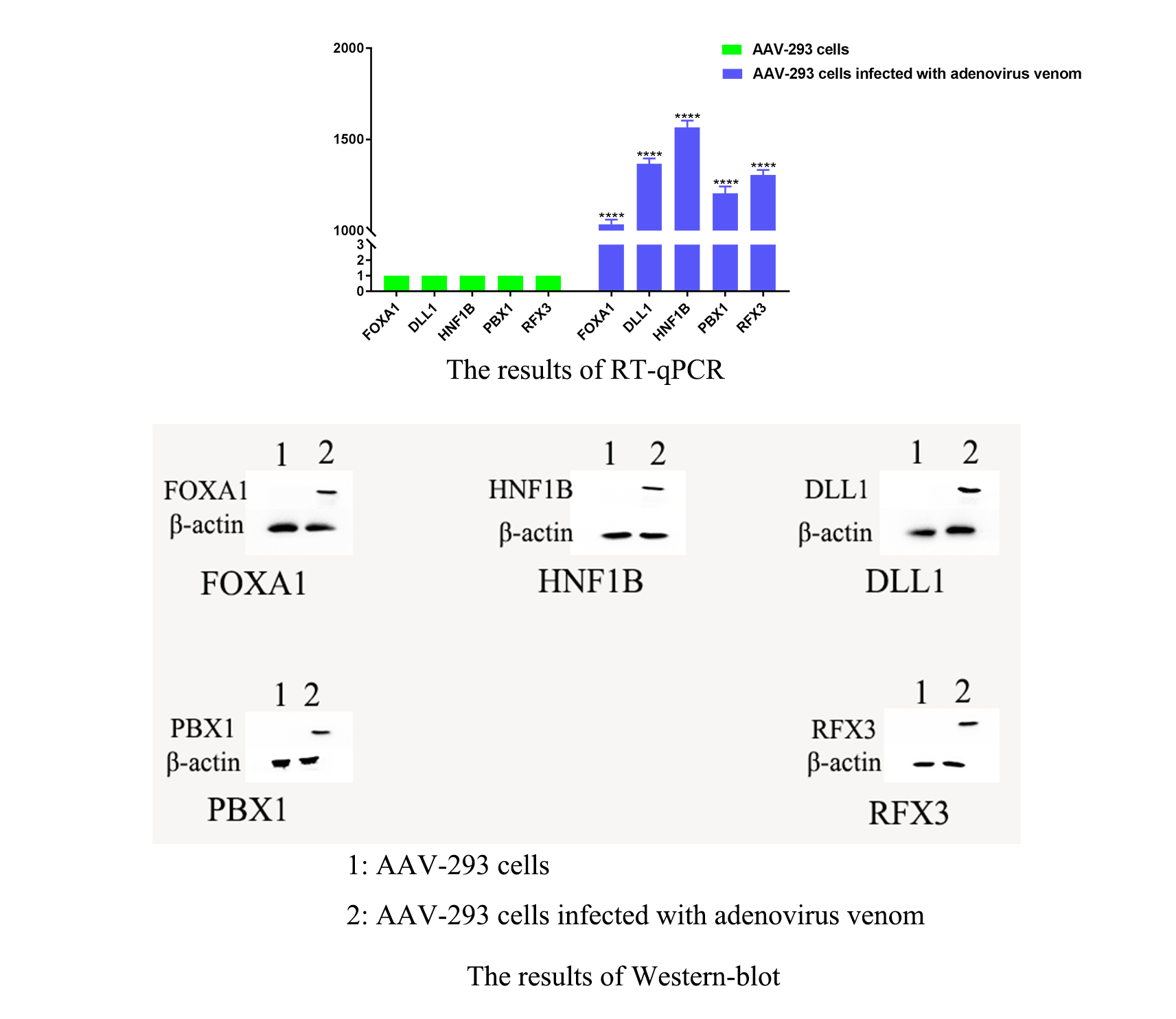

Supplement: Supplementary Material 1 — Five types of procedures. [file Data_Sheet_1.zip › Supplement 17.tif]

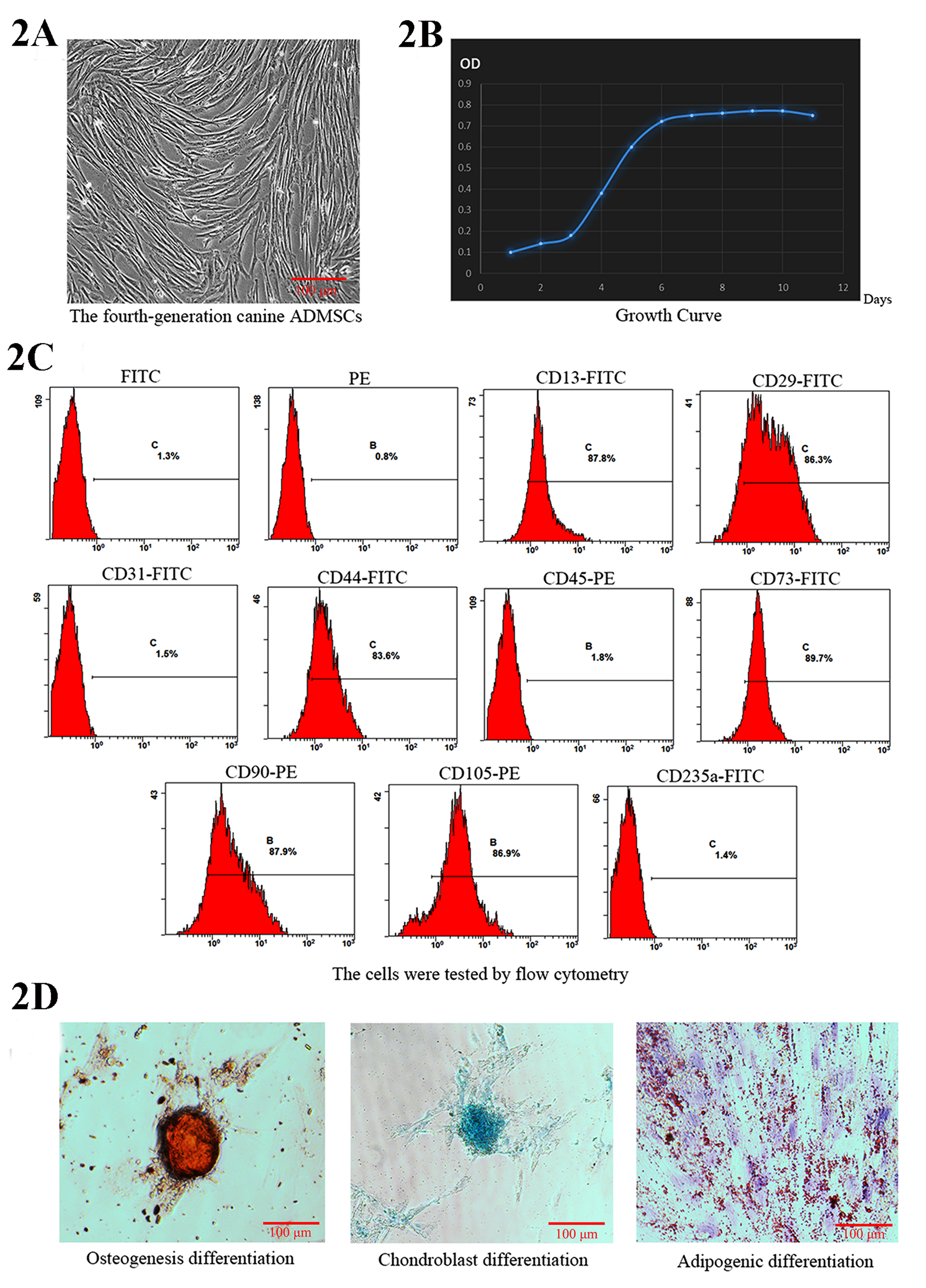

Supplement: Supplementary Material 1 — Five types of procedures. [file Data_Sheet_1.zip › Supplement 2.tif]

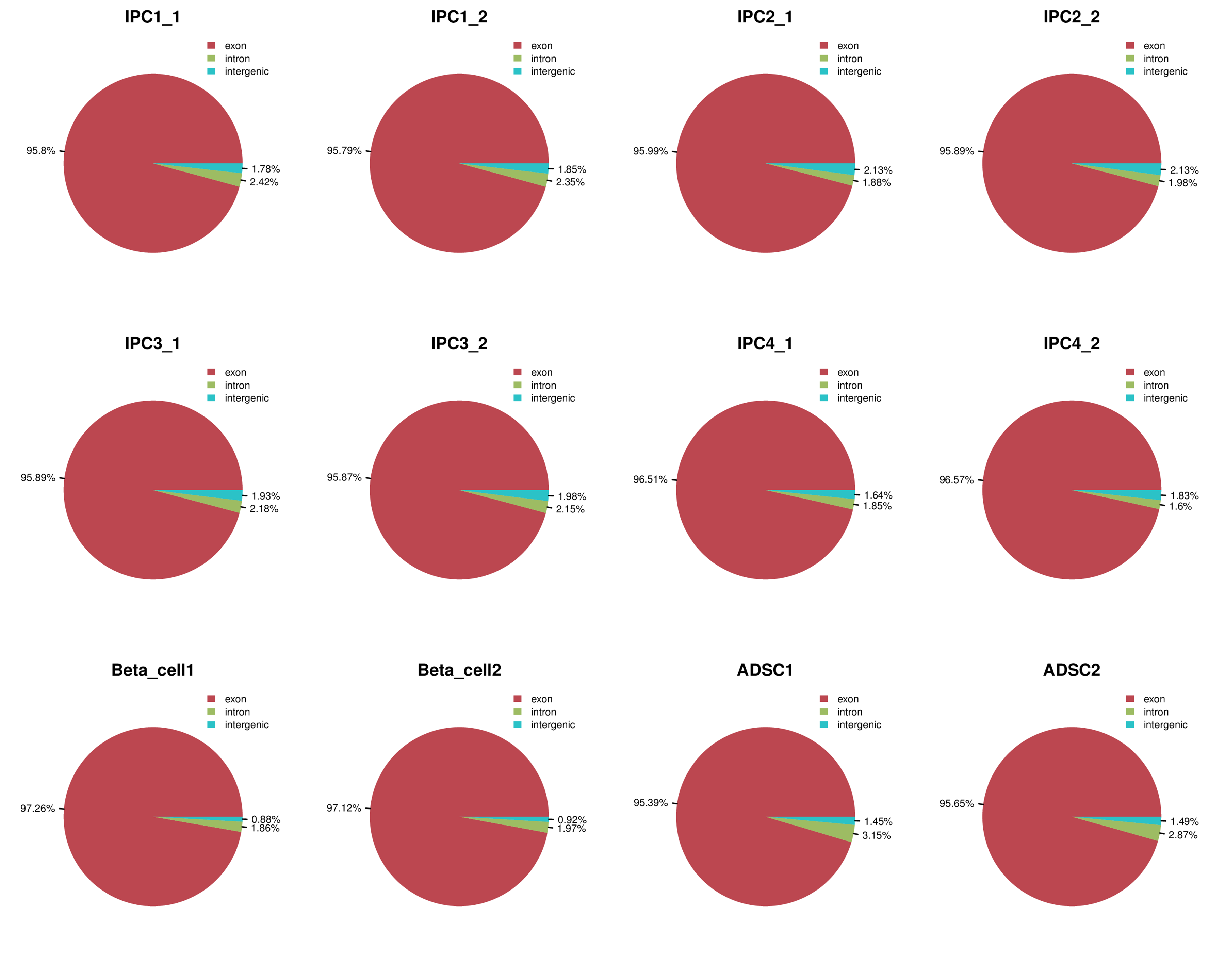

Supplement: Supplementary Material 1 — Five types of procedures. [file Data_Sheet_1.zip › Supplement 5.tif]

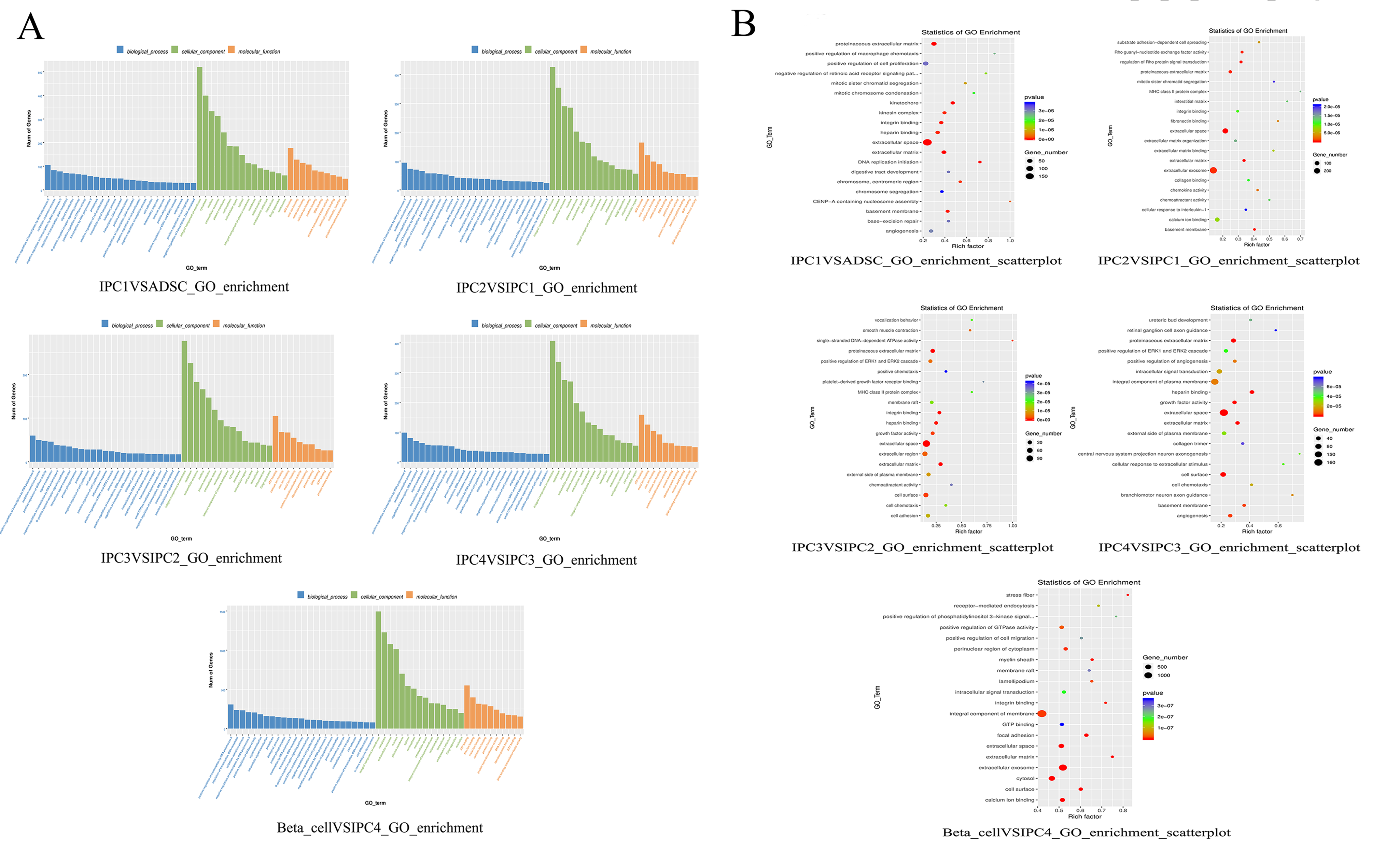

Supplement: Supplementary Material 1 — Five types of procedures. [file Data_Sheet_1.zip › Supplement 9.tif]
